# HDAC1 controls the generation and maintenance of effector-like CD8^+^ T cells during chronic viral infection

**DOI:** 10.1084/jem.20240829

**Published:** 2025-06-04

**Authors:** Ramona Rica, Monika Waldherr, Emi Miyakoda, Ana Patricia Kutschat, Marlene Schülein, Jing Zhang, Ricardo Alfredo Orbegozo-Medina, Lisa Sandner, Valentina Stolz, Darina Waltenberger, Thomas Krausgruber, Christoph Bock, Nicole Boucheron, Davide Seruggia, Wilfried Ellmeier, Shinya Sakaguchi

**Affiliations:** 1 Medical University of Vienna, Center for Pathophysiology, Infectiology and Immunology, Institute of Immunology, Vienna, Austria; 2 Department of Applied Life Sciences/Bioengineering/Bioinformatics, FH Campus Wien, Vienna, Austria; 3 CeMM Research Center for Molecular Medicine of the Austrian Academy of Sciences, Vienna, Austria; 4 Medical University of Vienna, Center for Medical Data Science, Institute of Artificial Intelligence, Vienna, Austria; 5 St. Anna Children’s Cancer Research Institute, Vienna, Austria

## Abstract

CD8^+^ T cell exhaustion is a complex process involving the differentiation of persistently activated CD8^+^ T cells into functionally distinct cell subsets. Here, we investigated the role of the key epigenetic regulator histone deacetylase 1 (HDAC1) in the differentiation of exhausted T (Tex) cells during chronic viral infection. We uncovered that HDAC1 controls the generation and maintenance of effector-like CX3CR1^+^ Tex cells in a CD8^+^ T cell–intrinsic manner. Deletion of HDAC1 led to expansion of an alternative Tex subset characterized by high expression of T cell exhaustion markers, and this was accompanied by elevated viremia. HDAC1 bound to and facilitated an open chromatin state of effector-like signature gene loci in progenitor Tex cells, thereby priming cell fate specification toward the CX3CR1^+^ Tex subset. Our study uncovers a selective role for HDAC1 in CX3CR1^+^ Tex subset differentiation, which is essential for controlling viral load during chronic infection.

## Teaser

Rica et al. demonstrate that HDAC1 promotes an open chromatin state at effector-like signature gene loci in exhausted T cell progenitors and thereby primes cell fate specification toward effector-like exhausted T cells, which is essential for controlling viral load during chronic infection.

## Graphical Abstract

**Figure:**
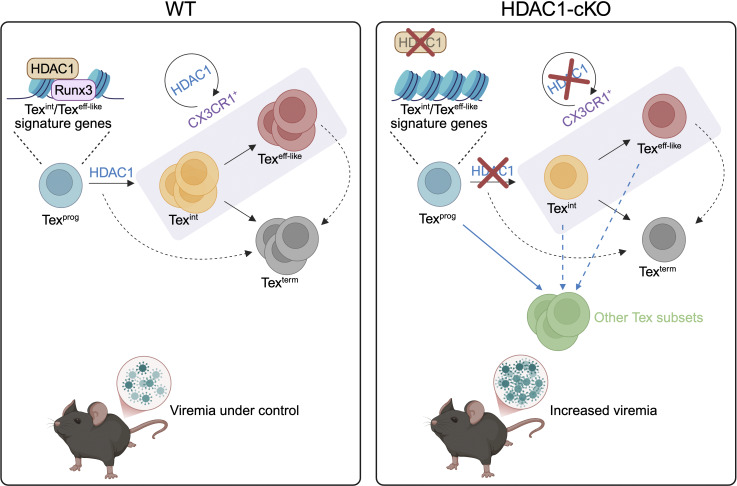

## Introduction

During chronic infection and cancer, CD8^+^ T cells display progressive loss of effector function accompanied by enhanced expression of inhibitory receptors and poor memory recall responses (Blank et al., 2019; McLane et al., 2019). These functional impairments are widely known as T cell exhaustion and are postulated as an evolutionarily conserved adaptation mechanism to persistent antigen stimulation, thereby limiting immunopathology or autoreactivity (Kallies et al., 2020; Speiser et al., 2014). Despite their altered state, exhausted T (Tex) cells still provide some protection against viral replication or tumor growth (Jin et al., 1999; Johnston et al., 2014; Schmitz et al., 1999). It is now evident that T cell exhaustion is a unique state of T cell differentiation, which is epigenetically distinct from effector T cells and memory T cells generated upon acute infection or vaccination (Belk et al., 2022; Zebley et al., 2020). It is therefore essential to elucidate the role of epigenetic regulators governing T cell exhaustion.

Tex cells are a heterogeneous population composed of functionally distinct subsets, which reflects the complex state and regulation of T cell exhaustion (Chung et al., 2021; McLane et al., 2019; Sandu and Oxenius, 2023; Zander and Cui, 2023). Tex cells are compartmentalized into at least two major subsets: a self-renewing progenitor subset (Tex^prog^ subset), defined by high expression levels of the transcription factor (TF) T cell factor 1 (TCF1) as well as the surface markers Ly108 and CXCR5, and a terminally exhausted TCF1^lo^ subset (Tex^term^), which is continuously replenished by Tex^prog^ cells (He et al., 2016; Im et al., 2016; Utzschneider et al., 2016; Wu et al., 2016). Importantly, Tex^prog^ cells display a proliferation burst upon immune checkpoint blockades, making them an attractive and promising target for immunotherapies (Kurtulus et al., 2019; Miller et al., 2019; Sade-Feldman et al., 2018). Subsequent studies identified additional Tex subsets, and several groups have independently identified a CX3CR1^+^ effector-like subset. This population, designated Tex^eff-like^ subset, retains a certain degree of cytolytic activity and thereby plays a central role in controlling chronic viral infection and tumor growth (Beltra et al., 2020; Hudson et al., 2019; Raju et al., 2021; Zander et al., 2019). Of note, immune checkpoint blockades lead to the expansion of CX3CR1^+^ cells and correlate with the better prognosis for patients with metastatic melanoma and non-small cell lung cancer (Yamauchi et al., 2021; Yan et al., 2018). Tex^eff-like^ cells display a unique transcriptional and epigenetic profile distinguishing them from Tex^prog^ and Tex^term^ subsets and, similar to Tex^term^ cells, are derived from Tex^prog^ cells. However, although several differentiation trajectories between Tex^eff-like^ and Tex^term^ cells have been postulated (Daniel et al., 2022; Giles et al., 2022; Kasmani et al., 2023), the exact developmental paths remain to be clarified. Since Tex^eff-like^ cells display a unique epigenetic profile (Chen et al., 2021a;Daniel et al., 2022; Giles et al., 2022), a better understanding of epigenetic pathways controlling their differentiation might reveal novel therapeutic approaches for chronic infection and cancer (Kanev and Zehn, 2021; Zander and Cui, 2023).

One of the best-described epigenetic modifications is reversible acetylation of lysine residues on histones, mediated by histone acetyltransferases and histone deacetylases (HDACs). The HDAC family consists of 18 members, and several HDACs have important functions in T cells (Ellmeier and Seiser, 2018). HDAC1 in T cells is a key driver of autoimmune diseases. Deletion of HDAC1 in T cells (using *Cd4*-Cre; *Hdac1*^fl/fl^ x *Cd4*-Cre, designated as HDAC1-cKO) conveys resistance to experimental autoimmune encephalomyelitis (Göschl et al., 2018; Hamminger et al., 2021), collagen-induced arthritis (Göschl et al., 2020), and adoptive T cell transfer colitis (Hamminger et al., 2021), while HDAC1 restrains the pathogenic Th2 cells in allergic airways inflammation (Grausenburger et al., 2010; Khan et al., 2025). HDAC1 is also essential for efficient in vivo expansion and activation of CD8^+^ T cells in response to acute lymphocytic choriomeningitis virus (LCMV) infection (Tschismarov et al., 2014), suggesting a role for HDAC1 in antiviral immunity. However, the role of HDAC1 in the differentiation of Tex subsets remains unknown.

Here, we elucidated the role of HDAC1 in CD8^+^ T cell exhaustion. By using a well-established murine model of chronic viral infection, we observed that HDAC1 regulates the generation and maintenance of the CX3CR1^+^ Tex^eff-like^ cell subset in a T cell–intrinsic manner. Moreover, HDAC1 was essential for controlling viral load. Single-cell RNA sequencing (scRNA-seq) revealed that HDAC1 deletion deviates the differentiation of Tex^prog^ cells into an alternative cell subset enriched in exhaustion and cytolytic signatures. Mechanistically, as revealed by assay for transposase accessible chromatin with sequencing (ATAC-seq) and cleavage under targets and release using nuclease (CUT&RUN), HDAC1 promoted the generation of Tex^eff-like^ cell subsets by binding to and opening effector-like signature gene loci in Tex^prog^ cells to prime their expression. Together, our study demonstrates a specific function of HDAC1 in driving CX3CR1^+^ Tex^eff-like^ cell subset differentiation, which is essential for viral control during chronic infection. Thus, targeting and modulating HDAC1-regulated pathways in CD8^+^ T cells might be an exciting therapeutic strategy for controlling chronic viral infection.

## Results

### T cell–specific deletion of HDAC1 leads to impaired viral control

**Figure 1.**
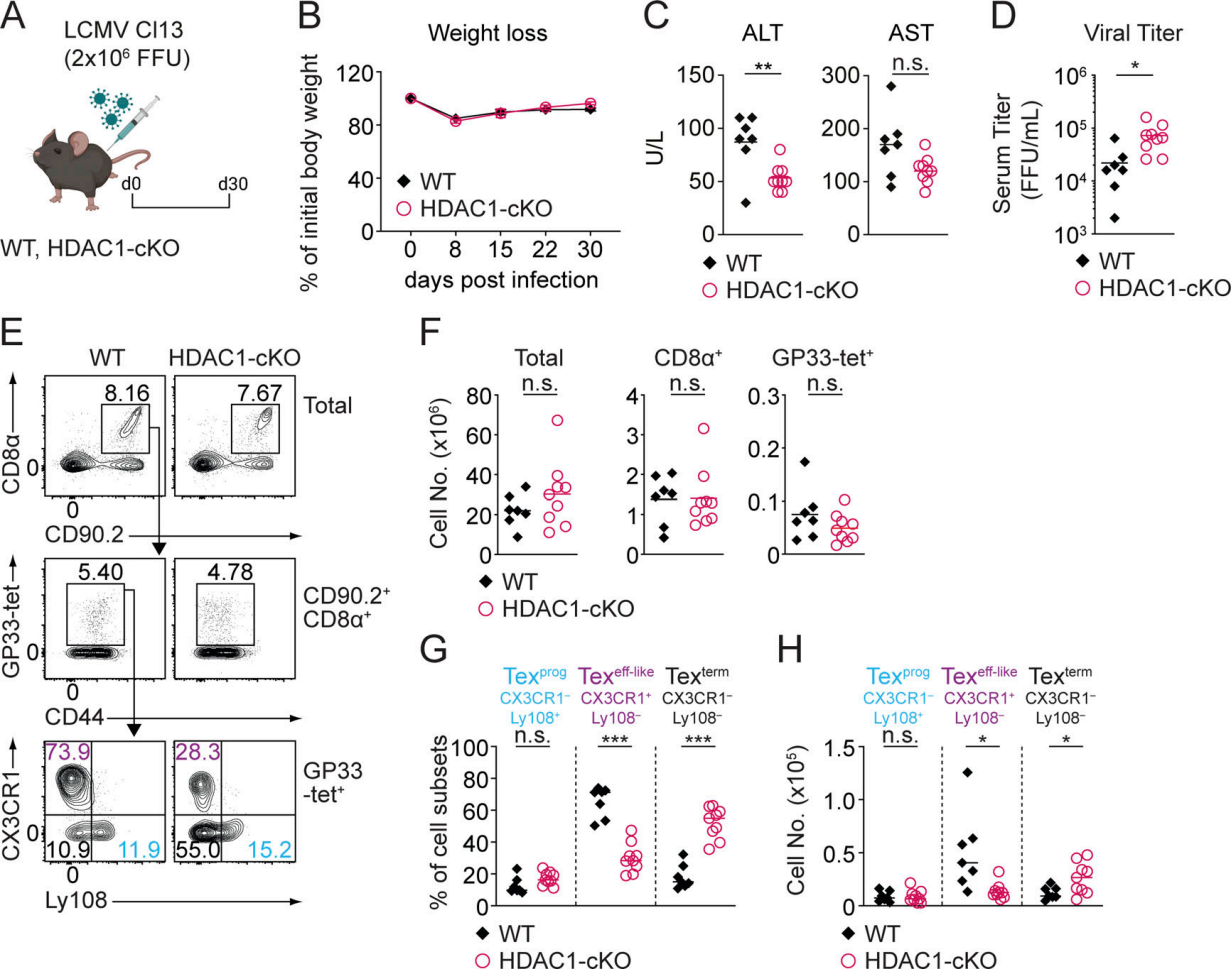
**Mice with a T cell–specific deletion of HDAC1 display impaired viral control during chronic infection. (A)** Schematic drawing of the experimental design. WT and HDAC1-cKO mice were infected with LCMV Cl13, and splenocytes were analyzed 30 days (d30) p.i. **(B)** Diagram shows weight loss as percentage of initial body weight during the course of infection. **(C)** Diagrams show the summary of alanine transaminase (ALT) and aspartate transaminase (AST) levels measured in units/liter (U/L) in the serum of infected mice on d30 p.i. **(D)** Diagram depicts viral titer in the serum of infected mice on d30 p.i. **(E)** Upper panel: Contour plots show the percentage of WT and HDAC1-cKO CD8^+^ T cells (CD90.2^+^CD8α^+^) from total splenocytes. Middle panel: CD8^+^ T cells were further gated on CD44^+^GP33-tet^+^ viral-specific Tex cells. Lower panel: Virus-specific Tex subsets were characterized by surface expression of CX3CR1 and Ly108: Tex^prog^ (CX3CR1^−^Ly108^+^) (blue), Tex^eff-like^ (CX3CR1^+^Ly108^−^) (purple), and Tex^term^ (CX3CR1^−^Ly108^−^) (black). **(F)** Diagrams show the summary of total numbers of total splenocytes (left), CD8α^+^ T cells (middle), and Tex cells (GP33-tet^+^) (right). **(G and H)** Diagrams show a summary of the percentages (G) and total numbers (H) of Tex subsets as depicted in E. Numbers in the plots (E) show the percentage of cells within the indicated regions. Data are representative (E) or show the summary (B–D and F–H) of seven to nine mice per genotype analyzed in two independent experiments. (C, D, and F–H) Horizontal bars indicate the mean. *P < 0.05; **P < 0.01; ***P < 0.001; n.s. P ≥ 0.05 (unpaired two-tailed Student’s *t* test).

To dissect the role of HDAC1 in T cell exhaustion, we induced chronic infection using the LCMV model in T cell–specific HDAC1-deficient (*Hdac1*^f/f^, *Cd4-Cre*) and corresponding WT control (*Hdac1*^f/f^) mice (hereafter referred to as HDAC1-cKO and WT mice, respectively) (Grausenburger et al., 2010). Specifically, we infected HDAC1-cKO and WT mice with the clone 13 strain of LCMV (LCMV Cl13) (Fig. 1 A), which results in chronic viral infection accompanied by a temporal weight loss and hepatitis driven by CD8^+^ T cells (Baazim et al., 2019; Bergthaler et al., 2007; Doherty et al., 1993; Moskophidis et al., 1993; Zinkernagel et al., 1986). Upon infection, HDAC1-cKO mice displayed a similar degree of a transient weight loss over a period of 4 wk in comparison to WT mice (Fig. 1 B). However, alanine aminotransferase levels were reduced in the serum of HDAC1-cKO mice at day 30 post infection (p.i.), and aspartate aminotransferase levels showed a similar tendency. This suggests ameliorated liver inflammation in the absence of HDAC1 (Fig. 1 C). Moreover, an elevated viremia in the serum in the absence of HDAC1 indicated its essential role in T cells for controlling antiviral responses (Fig. 1 D).

Based on the expression of CX3CR1 and Ly108, three exhausted CD8^+^ T cell subsets have been defined in established chronic infection (>3 wk p.i.): Tex^prog^ cells (Ly108^+^CX3CR1^−^), Tex^eff-like^ cells (Ly108^−^CX3CR1^+^), and Tex^term^ cells (Ly108^−^CX3CR1^−^) (Zander et al., 2019). Despite no alteration in the numbers of splenocytes or total and viral glycoprotein 33–41-specific (GP33-tet^+^) CD8^+^ T cells in the absence of HDAC1 (Fig. 1, E and F), a detailed flow cytometry analysis of CD8^+^ T cells revealed that lack of HDAC1 led to a reduction in Tex^eff-like^ cell frequencies and numbers (Fig. 1, E, G, and H). This was concurrent with an expansion of the Tex^term^ subset (Fig. 1, E, G, and H). Moreover, given their vital role in virus control (Hudson et al., 2019; Zander et al., 2019), the reduction of Tex^eff-like^ cells is in line with the observed increase in viremia in HDAC1-cKO mice (Fig. 1 D). Together, these data indicate that HDAC1 is essential for regulating Tex subset distribution and controlling viral load.

### HDAC1 is essential for early CX3CR1^+^ Tex cell differentiation

**Figure 2.**
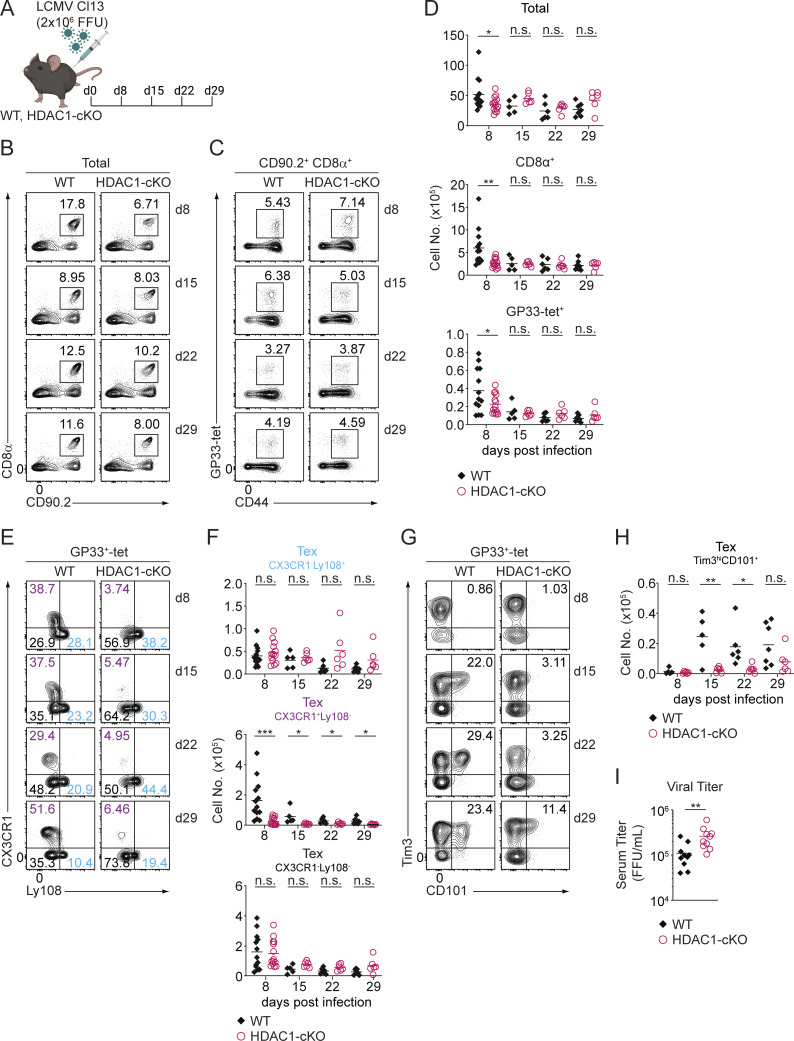
**Differentiation kinetics of Tex cell subsets during the course of chronic infection. (A)** Schematic drawing of the experimental design. WT and HDAC1-cKO mice were infected with LCMV Cl13, and splenocytes were analyzed on various time points (day [d]8, d15, d22, and d29) p.i. **(B)** Contour plots show the percentage of WT and HDAC1-cKO CD8^+^ T cells (CD90.2^+^CD8α^+^) from total splenocytes on various time points as depicted in A. **(C)** CD8^+^ T cells were further gated on viral-specific Tex cells (CD44^+^GP33-tet^+^). **(D)** Summary diagrams show total numbers of splenocytes (upper panel), CD8α^+^ T cells (middle), and Tex cells (GP33-tet^+^) (lower panel). **(E)** Representative plots show the distribution of Tex subsets on various time points of infection: early Tex^prog^ (CX3CR1^−^Ly108^+^) (blue), early Tex^eff-like^ (CX3CR1^+^Ly108^−^) (purple), and early Tex^nd^ (CX3CR1^−^Ly108^−^) (black). **(F)** Summary diagram depicts percentages of Tex subsets as shown in E. **(G)** Representative contour plots show Tim3 and CD101 expression on WT and HDAC1-cKO CD8^+^ T cells (pre-gated on CD90.2^+^CD8α^+^GP33^+^-tet) from splenocytes at different time points during infection. **(H)** Summary diagrams show the total numbers of Tim3^hi^CD101^+^ Tex cells at different time points as depicted in G. **(I)** Diagram depicts viral titer in the serum of infected mice on d8 p.i. Numbers in the plots (B, C, E, and G) show the percentage of cells within the indicated regions. Data are representative (B, C, E, and G) or show the summary (D, F, and H) of 5–14 mice per genotype/time point analyzed in three to five independent experiments or (I) eight mice per genotype from two independent experiments. *P < 0.05; **P < 0.01; ***P < 0.001; n.s. P ≥ 0.05 (unpaired two-tailed Student’s *t* test).

**Figure S1.**
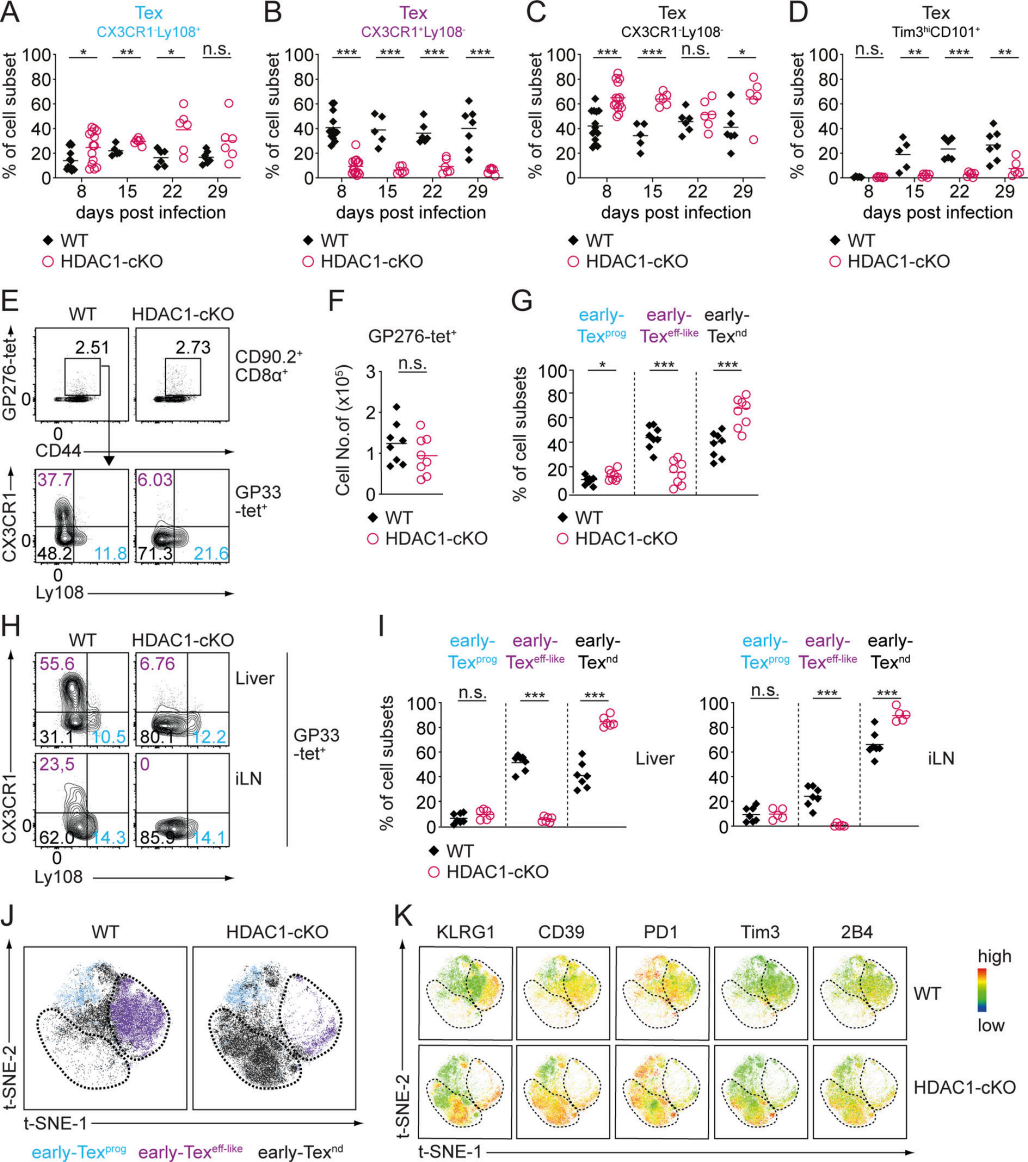
**Characterization of Tex cell subsets during the course of chronic infection. (A–C)** Diagrams show the summary of percentages of virus-specific Tex subsets characterized by surface expression of CX3CR1 and Ly108: Tex^prog^ (CX3CR1^−^Ly108^+^) (blue), Tex^eff-like^ (CX3CR1^+^Ly108^−^) (purple), and Tex^term^ (CX3CR1^−^Ly108^−^) (black) at day 8 (d8), d15, d22, and d30 p.i. **(D)** Diagram shows the summary of percentage of the double-positive (Tim3^hi^CD101^+^) T cell subset at different time points as described in A. **(E)** Contour plots show GP276-tet^+^ staining and CD44 expression (upper panel) and the distribution of Tex subsets in WT and HDAC1-cKO mice on d8 p.i.: early Tex^prog^ (CX3CR1^−^Ly108^+^) (blue), early Tex^eff-like^ (CX3CR1^+^Ly108^−^) (purple), and early Tex^nd^ (CX3CR1^−^Ly108^−^) (black) (lower panel). **(F)** Summary diagram shows total numbers of GP276-tet^+^ Tex cells. **(G)** Summary diagram depicts percentages of Tex subsets as shown in E. **(H)** Contour plots show distribution of GP33-tet^+^ Tex subsets (pre-gated on CD90.2^+^CD8^+^) in liver and inguinal LN (iLNs) on d8 p.i. **(I)** Diagrams show summary of the percentages of Tex subsets as depicted in H. **(J)** FlowJo generated t-SNE plots show clustering of WT and HDAC1-cKO Tex subsets on d8 p.i. **(K)** Relative expression of cell surface receptors KLRG1, CD39, PD1, Tim3, and 2B4 used as parameters for t-SNE dimensionality reduction as depicted in J. Numbers in the plots (E and H) show the percentage of cells within the indicated quadrants and regions. Data are representative (E and H) or show the summary (A–D) of 5–14 mice per genotype/time point analyzed in three to five independent experiments or (F, G, and I) five to eight mice per genotype from two independent experiments. Data show summary (J and K) of concatenated samples from four mice per genotype. *P < 0.05; **P < 0.01; ***P < 0.001; n.s. P ≥ 0.05 (unpaired two-tailed Student’s *t* test).

The emergence of Tex cells, based on transcriptional and chromatin accessibility profiling, has been observed as early as day 8 p.i. (Daniel et al., 2022; Giles et al., 2022; Yao et al., 2019). Having uncovered that HDAC1 deletion resulted in a reduction of Tex^eff-like^ cells 30 days p.i., we next determined the time point during infection at which Tex^eff-like^ cell differentiation is affected by HDAC1 deficiency. Since Tex^term^ cells (defined as Ly108^−^CX3CR1^−^ cells) were increased in HDAC1-cKO mice (Fig. 1, E, G, and H), we monitored the appearance of Tim3^hi^CD101^+^ Tex cells as an alternative approach of identifying Tex^term^ cells (Hudson et al., 2019). For this, we assessed the expansion kinetics of splenic CD8^+^ T cells as well as Tex subset distribution among virus-specific CD8^+^ T cells in LCMV Cl13–infected HDAC1-cKO and WT mice on day 8, 15, 22, and 29 p.i. (Fig. 2 A). This analysis revealed that lack of HDAC1 led to a reduction in the numbers of CD8^+^ and GP33-tet^+^ CD8^+^ T cells 8 days p.i. (Fig. 2, B–D). Moreover, whereas WT mice displayed a strong induction of CX3CR1^+^ cells among GP33-tet^+^ CD8^+^ T cells from day 8 p.i. on as previously reported (Daniel et al., 2022; Zander et al., 2019), the percentages and numbers of this subset were much lower in HDAC1-cKO mice (Fig. 2, E and F; and Fig. S1, A–C). Furthermore, the number of Tim3^hi^CD101^+^ Tex cells, which emerged from day 15 p.i. in WT mice, was reduced at day 15 and 22 p.i. in the mutant mice (Fig. 2, G and H; and Fig. S1 D). Thus, HDAC1 deletion impaired the differentiation of Tex cells (characterized as CX3CR1^+^ or Tim3^hi^CD101^+^ cells) from the onset of chronic viral infection. Ly108^+^CX3CR1^−^ cells acquire a certain degree of Tex^prog^ features as early as 8 days p.i. (Daniel et al., 2022; Giles et al., 2022; Utzschneider et al., 2020; Zander et al., 2019). Moreover, fate-mapping experiments demonstrated that CX3CR1^+^ Tex cells emerging around 8 days p.i. later give rise to a substantial proportion of a Tex^eff-like^ cell subset (Raju et al., 2021). Hence, we refer to these two subsets on day 8 as early Tex^prog^ cells and early Tex^eff-like^ cells, respectively, throughout the manuscript. However, given that virtually no Tex^term^ cells emerge on day 8 p.i. (Fig. 2, G and H; and Fig. S1 D) (Daniel et al., 2022; Giles et al., 2022; Utzschneider et al., 2020; Zander et al., 2019), we termed the Ly108^−^CX3CR1^−^ subset, with no defined characteristics (nd), early Tex^nd^ cells.

Having revealed an essential role for HDAC1 in the generation of splenic early Tex^eff-like^ cells, we further characterized this subset at day 8 p.i. A comparable reduction of the early Tex^eff-like^ cell subset in HDAC1-deficient CD8^+^ T cells specific for the viral glycoprotein 276–286 (GP276-tet^+^) indicated that the altered differentiation of Tex subsets occurs independently of TCR specificity (Fig. S1, E–G). Moreover, a severely diminished early Tex^eff-like^ cell population was observed in other lymphoid (i.e., inguinal LNs) as well as nonlymphoid (i.e., liver) organs in HDAC1-cKO mice (Fig. S1, H and I), indicating phenotypic alterations of Tex subsets across many tissues. Finally, HDAC1-cKO mice displayed a higher serum viremia on day 8 p.i. when compared with WT mice (Fig. 2 I). This indicates that early Tex^eff-like^ cells play an essential role in controlling viral replication, similar to Tex^eff-like^ cells that emerge at later time points during infection.

**Figure 3.**
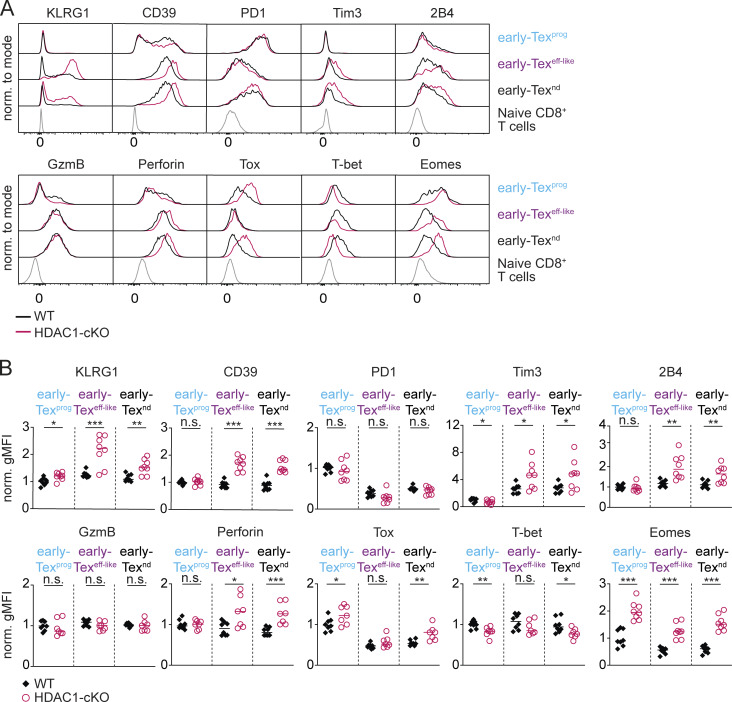
**HDAC1 is essential for CX3CR1** ^**+**^ **Tex cell differentiation at the onset of chronic viral infection. (A)** WT and HDAC1-cKO mice were infected with LCMV Cl13, and splenocytes were analyzed 8 days (d8) p.i. Histograms show the expression of the surface receptors KLRG1, CD39, PD1, Tim3, and 2B4 (upper panel) as well as the cytolytic molecules GzmB and perforin together with the TFs Tox, T-bet, and Eomes (lower panel) in all Tex subsets (pre-gated on CD8α^+^CD90.2^+^CD44^+^GP33-tet^+^ cells) on d8 p.i. The bottom row of histograms shows the expression levels of each molecule in splenic naive CD8^+^ T cells from non-infected WT mice. **(B)** Diagrams display normalized geometric mean fluorescence intensity (gMFI; average WT early Tex^prog^ expression set to 1) of all markers depicted in A. Data are representative (A) or show the summary (B) of eight mice per genotype analyzed in two independent experiments. **(B)** Horizontal bars indicate the mean. *P < 0.05; **P < 0.01; ***P < 0.001; n.s. P ≥ 0.05 (unpaired two-tailed Student’s *t* test).

In addition to the markers Ly108 and CX3CR1, Tex cells also display specific and distinct expression patterns of inhibitory receptors, effector molecules, and TFs (Beltra et al., 2020; Hudson et al., 2019; Zander et al., 2019). Additional immunophenotyping revealed that the expression levels of inhibitory receptors, such as Tim3 and 2B4, as well as of CD39, an ecto-ATPase linked with T cell exhaustion, were increased in HDAC1-cKO early Tex^eff-like^ and early Tex^nd^ cells (Fig. 3, A and B). This was accompanied by an elevated expression of thymocyte selection-associated HMG box (Tox), a central TF initiating T cell exhaustion (Fig. 3, A and B) (Alfei et al., 2019; Khan et al., 2019; Scott et al., 2019; Seo et al., 2019; Yao et al., 2019). Moreover, lack of HDAC1 resulted in elevated eomesodermin (Eomes) expression levels, concurrent with the downmodulation of T-box TF 21 (T-bet) expression (Fig. 3, A and B). Similar expression patterns of Eomes and T-bet are linked to terminal exhaustion (McLane et al., 2021; Zehn et al., 2022). Besides the increased expression of proteins linked to an exhaustion state, HDAC1-deficient Tex cells displayed enhanced expression of killer cell lectin-like receptor G1 (KLRG1), a canonical marker for effector T cells (Fig. 3, A and B) (Chen et al., 2019; Chung et al., 2021; Martin and Badovinac, 2018). In addition, while granzyme B (GzmB) expression was unchanged, perforin expression was higher in HDAC1-deficient early Tex^eff-like^ and early Tex^nd^ cells (Fig. 3, A and B), indicating that certain effector features are enhanced upon lack of HDAC1. To gain further insight into the impact of HDAC1 deletion on the heterogeneity and subset composition of Tex cells, we applied t-distributed Stochastic Neighbor Embedding (t-SNE) dimensionality reduction to our flow cytometry data. While a population corresponding to early Tex^eff-like^ cells was easily detectable in WT mice, deletion of HDAC1 led to an almost complete loss of this subset (Fig. S1 J, encircled purple population). Instead, a unique population (encircled black population) co-expressing inhibitory receptors and KLRG1 at high levels emerged in HDAC1-cKO mice (Fig. S1, J and K). Taken together, these results highlight an essential role of HDAC1 in promoting the generation of early Tex^eff-like^ cells.

### HDAC1 controls early Tex^eff-like^ subset differentiation in a CD8^+^ T cell–intrinsic manner

**Figure 4.**
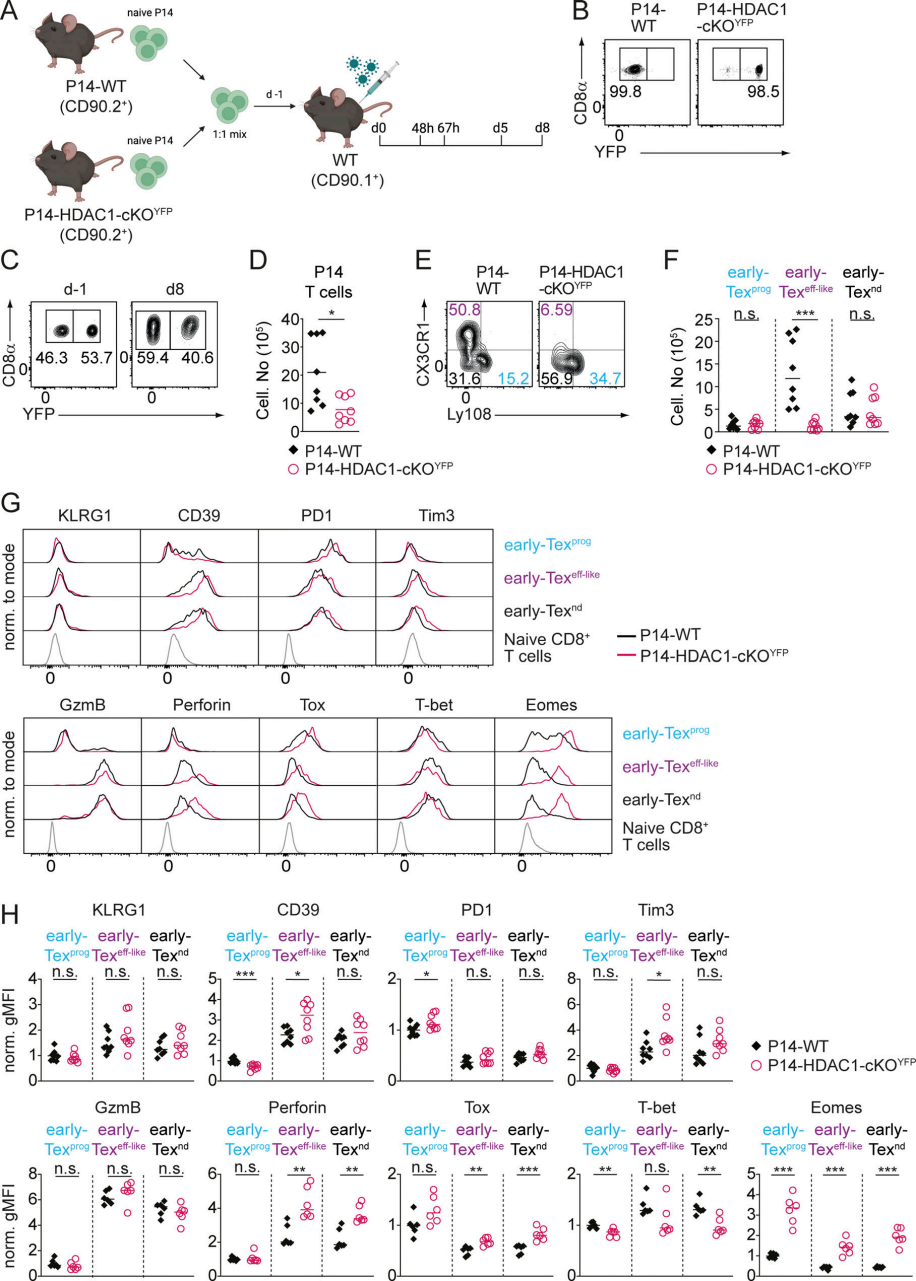
**HDAC1 controls early Tex** ^**eff-like**^ **subset differentiation in a CD8** ^**+**^ **T cell–intrinsic manner. (A)** Scheme of the experimental design. Naïve P14-WT and P14-HDAC1-cKO^YFP^ cells (CD90.2^+^) were isolated and transferred into congenic CD90.1^+^ recipient mice one day prior (d-1) to LCMV Cl13 infection. The differentiation kinetics and the composition of Tex subsets were determined at various time points after infection (0 h, 48 h, 67 h, d5, and d8). **(B)** Contour plots show YFP and CD8α expression in freshly isolated naïve P14-WT and P14-HDAC1-cKO^YFP^ cells before adoptive transfer. **(C)** Contour plots show the numbers of YFP^−^ P14-WT and YFP^+^ P14-HDAC1-cKO^YFP^ cells on the day of transfer (d-1) and on d8 p.i. **(D)** Diagram shows the summary of the percentages of transferred P14-WT and P14-HDAC1-cKO^YFP^ cells on d8 p.i. as depicted in C. **(E)** Contour plots show the distribution of Tex subsets on d8 p.i.: Tex^prog^ (CX3CR1^−^Ly108^+^) (blue), early Tex^eff-like^ (CX3CR1^+^Ly108^−^) (purple), and early Tex^nd^ (CX3CR1^−^Ly108^−^) (black) within transferred P14 cells. **(F)** Diagram shows the summary of the numbers of Tex subsets as depicted in E. **(G)** Histograms show the expression of the cell surface molecules KLRG1, CD39, PD1, and Tim3 (upper panel) and cytolytic molecules GzmB and perforin, as well as the TFs Tox, T-bet, and Eomes (lower panel), in all Tex subsets of adoptively transferred P14-WT or P14-HDAC1-cKO^YFP^ cells (pre-gated on CD8α^+^CD90.2 cells) on day 8 p.i. The bottom row of histograms shows the expression levels of each molecule in splenic naive CD8^+^ T cells from non-infected WT mice. **(H)** Diagrams show normalized geometric mean fluorescence intensity (gMFI; average WT early Tex^prog^ expression set to 1) of all markers depicted in G. Numbers in the plots (B, C, and E) show the percentage of cells within the indicated regions. Data shown are representative (B, C, E, and G) or show the summary (D, F, and H) of eight mice per genotype analyzed in two independent experiments (D and F) or eight mice per genotype analyzed in two independent experiments (H). **(D, F, and H)** Horizontal bars indicate the mean. *P < 0.05; **P < 0.01; ***P < 0.001; n.s. P ≥ 0.05 (unpaired two-tailed Student’s *t* test was used).

**Figure S2.**
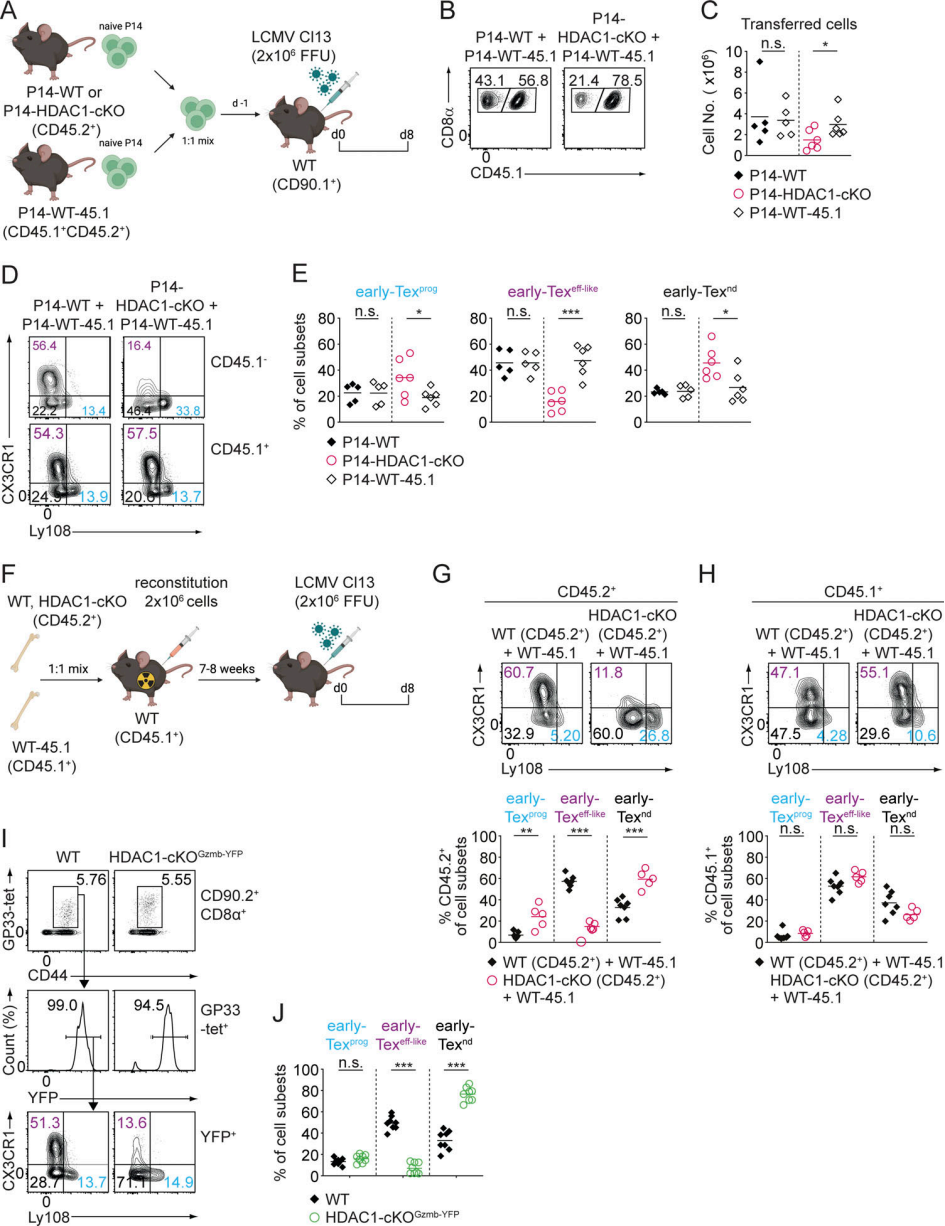
**HDAC1 controls Tex subset differentiation in a T cell–intrinsic manner. (A)** Schematic drawing of the experimental design. Naïve P14-WT or P14-HDAC1-cKO cells (CD45.2^+^) were mixed in a 1:1 ratio with P14-WT-45.1 cells (CD45.1^+^CD45.2^+^) and adoptively transferred into WT (CD90.1^+^) recipient mice one day prior infection. Recipient mice were infected with LCMV Cl13, and splenocytes were analyzed on day 8 (d8) p.i. **(B)** Contour plots depict the cellular distribution of adoptively transferred P14 cells within the recipient mice on d8 p.i. **(C)** Diagram shows the total numbers of adoptively transferred cells as depicted in B. **(D)** Contour plots depict the distribution of Tex subsets (early Tex^prog^, CX3CR1^−^Ly108^+^, blue; early Tex^eff-like^, CX3CR1^+^Ly108^−^, purple; and early Tex^nd^, CX3CR1^−^Ly108^−^, black) within adoptively transferred P14-WT and P14-HDAC1-cKO (CD45.1^−^; upper panel) or P14-WT-CD45.1 (CD45.1^+^; lower panel) cell compartment. **(E)** Diagrams show the percentage of Tex subsets within the different cell compartments, as depicted in D. **(F)** Schematic drawing of the experimental design. BM from either WT (CD45.2^+^) or HDAC1-cKO (CD45.2^+^) mice was mixed with BM from WT CD45.1^+^ mice at a 1:1 ratio and transferred into irradiated CD45.1^+^ recipient mice. Reconstituted mice were infected with LCMV Cl13 7–8 wk after BM transplantation. Eight days later (d8) splenocytes were analyzed by flow cytometry. **(G)** Contour plots (upper panel) and diagrams (lower panel) show the distribution and percentages of Tex subsets on d8 p.i. within the CD45.2^+^ (WT or HDAC1-cKO) cell compartment: early Tex^prog^ (CX3CR1^−^Ly108^+^) (blue), early Tex^eff-like^ (CX3CR1^+^Ly108^−^) (purple), and early Tex^nd^ (CX3CR1^−^Ly108^−^) (black). Cells were pre-gated on CD90.2^+^CD8α^+^GP33-tet^+^CD44^+^ cells. **(H)** Contour plots (upper panel) and diagrams (lower panel) show the distribution and percentages of Tex subsets on d8 p.i. within the CD45.1^+^ (WT) cell compartment. **(I)** Flow cytometry analysis showing WT and HDAC1-cKO^Gzmb-YFP^ GP33-tet^+^ T cells (pre-gated on CD90.2^+^CD8α^+^) from total splenocytes (upper panel) on d8 p.i. Viral-specific exhausted CD8^+^ T (Tex) cells (GP33-tet^+^) were further gated based on their YFP expression (i.e., activated T cells) (middle panel). CX3CR1 and Ly108 expression on YFP^+^ Tex subsets is shown in the lower panel: early Tex^prog^ (CX3CR1^−^Ly108^+^) (blue), early Tex^eff-like^ (CX3CR1^+^Ly108^−^) (purple), and early Tex^nd^ (CX3CR1^−^Ly108^−^) (black). **(J)** Diagrams show the summary of percentages of Tex subsets as depicted in I. Numbers in the plots (B, D, and G–I) show the percentage of cells within the indicated regions. Data are representative (B, D, and G–I) or show the summary (C, E, and J) of five to six mice (C and E), five to nine mice (G and H), or eight mice (J) per genotype analyzed in two independent experiments. *P < 0.05; **P < 0.01; ***P < 0.001; n.s. P ≥ 0.05 (unpaired two-tailed Student’s *t* test).

The formation of Tex^eff-like^ cells requires help from CD4^+^ T cells that secrete IL-21 (Raju et al., 2021; Zander et al., 2019). Since *Hdac1* is deleted both in CD4^+^ and CD8^+^ T cells in HDAC1-cKO mice (owing to the expression of Cre recombinase from the late double-negative thymocyte developmental stage onward in the *Cd4*-Cre deleter line) (Lee et al., 2001), alterations in the subset distribution of Tex cells might be due to a dysfunction of either CD4^+^, CD8^+^ T cells, or even both. In addition, it is possible that HDAC1 deletion leads to developmental defects in thymocytes that result in impaired early Tex^eff-like^ cell differentiation. Moreover, the increased viremia observed in HDAC1-cKO mice might feedback on early Tex^eff-like^ cell differentiation. To distinguish among these possibilities, we employed several experimental approaches. Firstly, we performed adoptive transfer experiments with CD8^+^ T cells that express the transgenic P14 TCR, recognizing the viral GP33 peptide presented by H2D^b^ (Pircher et al., 1989). We crossed WT and HDAC1-cKO mice with P14 TCR transgenic mice and additionally introduced a *Rosa26*-STOP-EYFP reporter allele (Srinivas et al., 2001) (hereafter referred to as P14-WT and P14-HDAC1-cKO^YFP^ mice, respectively) (Fig. 4 A). This allows the tracking of YFP^+^ P14-HDAC1-cKO^YFP^ T cells due to Cre-mediated deletion of the STOP cassette (Fig. 4 B). Naïve P14-WT (CD90.2^+^) and P14-HDAC1-cKO^YFP^ (CD90.2^+^YFP^+^) cells were mixed at a 1:1 ratio and adoptively co-transferred into WT CD90.1^+^ recipient mice (Fig. 4 C). On the following day, recipient mice were infected with LCMV Cl13, and the differentiation of Tex cells was analyzed 8 days p.i. (Fig. 4 A). Consistent with the reduced number of virus-specific CD8^+^ T cells observed in HDAC1-cKO mice (Fig. 2, C and D), the number of P14-HDAC1-cKO^YFP^ cells was reduced in this competitive setting (Fig. 4, C and D). Moreover, P14-HDAC1-cKO^YFP^ cells “phenocopied” the distribution of Tex subsets that has been observed in HDAC1-cKO mice (Fig. 2, E and F), showing reduced numbers of early Tex^eff-like^ cells (Fig. 4, E and F). Of note, additional co-transfer experiments using congenically marked CD45.1^+^ or CD45.2^+^ WT and HDAC1-deficient donor cells (instead of YFP^–^ and YFP^+^ cells), revealed the same alterations in the mutant cells (Fig. S2, A–E). This indicates that the phenotypic changes in HDAC1-cKO CD8^+^ T cells were not due to the potential immunogenicity of the YFP protein possibly resulting in rejection of certain cell subsets. Secondly, we also generated mixed bone marrow (BM) chimeric mice, where WT (CD45.2^+^) or HDAC1-cKO (CD45.2^+^) BM cells were mixed at a 1:1 ratio with congenically distinguishable WT (CD45.1^+^) BM cells (Fig. S2 F). Following reconstitution of irradiated recipients (CD45.1^+^) and subsequent infection, we observed reduced frequencies of the early Tex^eff-like^ cell subset within the reconstituted CD45.2^+^ HDAC1-deficient but not CD45.2^+^ WT and CD45.1^+^ compartment (Fig. S2, G and H). This was congruent with results obtained from the adoptive T cell co-transfer experiments. Finally, to exclude that a potential developmental defect due to HDAC1 deletion contributed to changes in Tex subsets, we specifically deleted HDAC1 in mature CD8^+^ T cells and only after their activation. For this, we generated HDAC1-deficient mice on a *Gzmb*-Cre background and then introduced a *Rosa26*-STOP-YFP allele to monitor Cre activity (*Hdac1*^f/f^, *Gzmb*-Cre, and *Rosa*26-STOP-YFP; hereafter referred to as HDAC1-cKO^Gzmb-YFP^ mice). Since *Gzmb*-Cre deletes in activated T cells (Jacob and Baltimore, 1999), any alterations seen in these mice are due to a role of HDAC1 during or after T cell activation and Tex cell differentiation. We infected HDAC1-cKO^Gzmb-YFP^ mice and WT^Gzmb-YFP^ control mice (i.e., *Hdac1*^+/+^, *Gzmb*-Cre, and *Rosa26*-STOP-YFP) with LCMV Cl13 and immunophenotyped virus-specific CD8^+^ T cells 8 days p.i. (Fig. S2, I and J). Similar to our observation made in HDAC1-cKO mice, the CX3CR1^+^Ly108^–^ early Tex^eff-like^ subset was reduced in HDAC1-cKO^Gzmb-YFP^ mice (Fig. S2, I and J). Together, these results clearly indicate that HDAC1 controls the generation of early Tex^eff-like^ subsets in a CD8^+^ T cell–intrinsic manner and independently of antigen levels (i.e., viral titer).

### HDAC1-deficient early Tex cells display intact cytolytic activity in vitro

**Figure S3.**
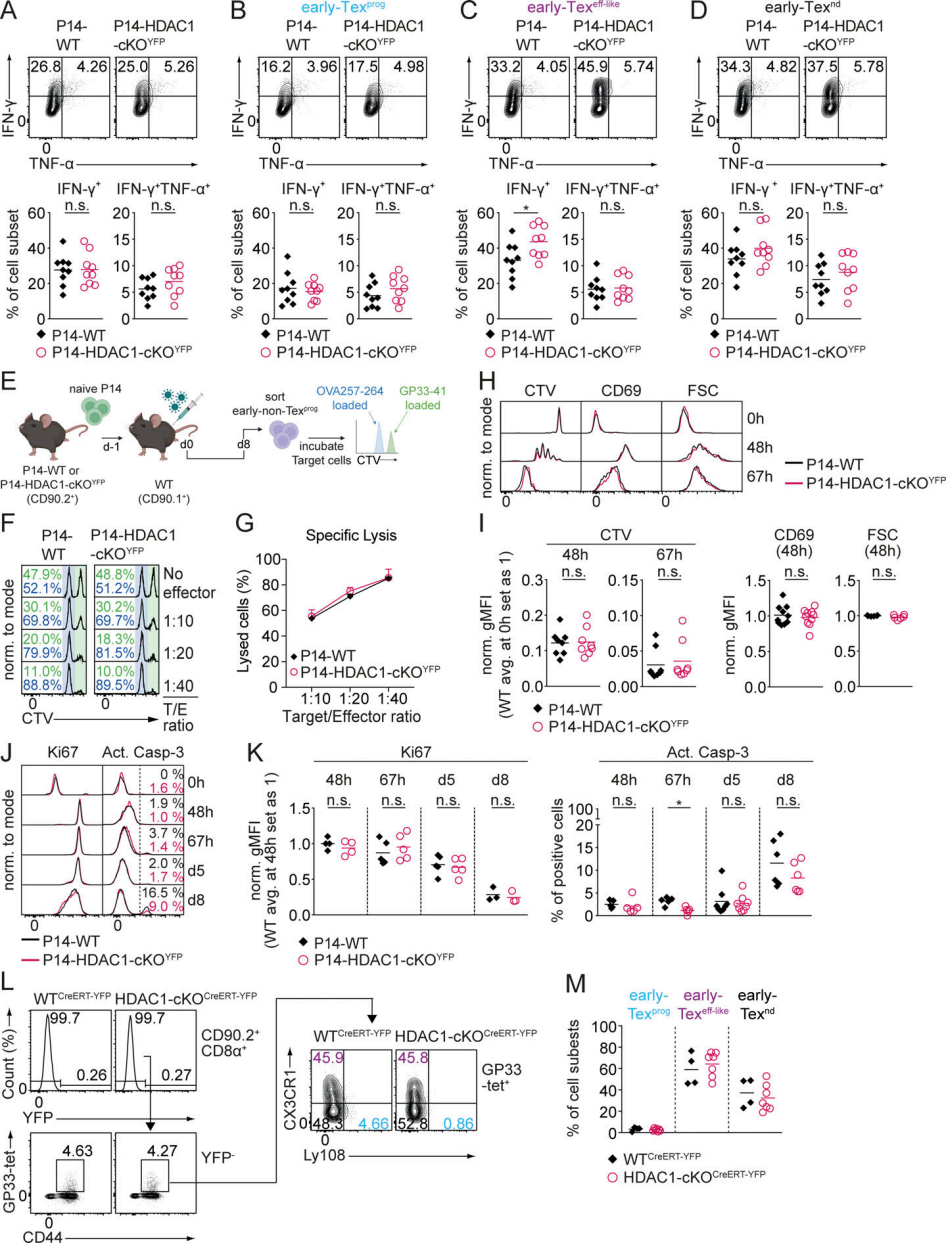
**Characterization of HDAC1-cKO Tex effector functions. (A–D)** Upper panels: Representative contour plots show expression of IFN-γ and TNF-α in total P14-WT or P14-HDAC1-cKO^YFP^ cells (A), early Tex^prog^ cells (CX3CR1^−^Ly108^+^; B), early Tex^eff-like^ cells (CX3CR1^+^Ly108^−^; C), and early Tex^nd^ cells (CX3CR1^−^Ly108^−^; D) on day 8 (d8) p.i. Cells were pre-gated on CD90.2^+^CD8^+^GP33-tet^+^ cells. Lower panels: Diagram shows percentage of IFN-γ as well as IFN-γ/TNF-α–expressing subsets of P14-WT and P14-HDAC1-cKO^YFP^ cells. **(E)** Schematic drawing of the experimental design. Naïve P14-WT or P14-HDAC1-cKO^YFP^ cells (CD90.2^+^) were adoptively transferred into WT (CD90.1^+^) recipient mice one day prior to infection. Recipient mice were infected (d0), and on d8 p.i., early non-Tex^prog^ (Tim3^+^) cells were sorted and used for in vitro cytotoxic killing assays by co-culturing them with CellTrace Violet (CTV)–labeled OVA257–264 (nonrelevant peptide) or GP33–41- (relevant peptide) pulsed EL-4 target cells. **(F)** Histograms show CTV dilution in nonrelevant pulsed (low CTV concentration—blue rectangle) or relevant pulsed (high CTV concentration—green rectangle) target cells after co-culture with WT or HDAC1-cKO^YFP^ non-Tex^prog^ cells at different target (T) to effector (E) cell ratios. **(G)** Diagram shows the percentage of cell-specific lysis by WT or HDAC1-cKO^YFP^ non-Tex^prog^ cells as depicted in F. **(H)** Histograms show cell proliferation (CTV), activation marker CD69 expression, and forward scatter (FSC) of P14-WT and P14-HDAC1-cKO^YFP^ cells at 48 and 67 h p.i. **(I)** Diagrams show relative expression levels (gMFI) of markers depicted in H. Expression levels in P14-WT cells at time point 0 h (for CTV) or 48 h (for CD69 and FSC) were set as 1. **(J)** Histograms show the expression of the proliferation marker Ki67 and the apoptosis marker active caspase-3 (Act. Casp-3) in P14-WT and P14-HDAC1-cKO^YFP^ cells at the indicated time points of infection (0 h, 48 h, 67 h, d5, and d8). **(K)** Diagrams show relative expression levels (gMFI) of Ki67 or percentages of Act. Casp-3^+^ cells depicted in J. Expression levels of Ki67 in P14-WT cells at time point 48 h was set as 1. **(L)** Histograms or contour plots show the percentages of YFP expression in CD90.2^+^CD8α^+^ cells (upper panel), GP33-tet^+^ staining and CD44 expression (pre-gated on YFP^–^ cells; middle panel), and the distribution of GP33-tet^+^ Tex subsets in WT^CreERT2^ and HDAC1-cKO ^CreERT2^ mice on d8 p.i before tamoxifen administration: early Tex^prog^ (CX3CR1^−^Ly108^+^) (blue), early Tex^eff-like^ (CX3CR1^+^Ly108^−^) (purple), and early Tex^nd^ (CX3CR1^−^Ly108^−^) (black) (lower panel). **(M)** Diagrams show the summary of percentages of Tex subsets as depicted in K. Numbers in the histograms (F and J) or plots (A–D, and L) show the percentage of cells in the indicated regions. Data are representative (A–D, F, H, J, and L) or show the summary (A–D, G, I, K, and M) of nine mice per genotype (A–D), four replicates, each consisting of two mice per genotype (G), 6–10 (I), 3–8 (K), or 4–7 (M) mice per genotype analyzed from 3 (A–D), 2 (G), 3–5 (I), 2–4 (K), or 2 (M) independent experiments. **(A–D, I, K, and M) **Horizontal bars indicate the mean. *P < 0.05; n.s. P ≥ 0.05 (unpaired two-tailed Student’s *t* test). gMFI, geometric mean fluorescence intensity.

Next, we characterized effector functions of P14-HDAC1-cKO^YFP^ cells 8 days p.i. in the adoptive T cell co-transfer setting. The examination of immunoregulatory and effector protein expression revealed that donor P14-HDAC1-cKO^YFP^ cells displayed essentially the same alterations as the ones observed on CD8^+^ T cells in infected HDAC1-cKO mice (Fig. 4, G and H). This underscores the CD8^+^ T cell–intrinsic role of HDAC1 for early Tex cell differentiation. Furthermore, the expression of IFN-γ and TNF-α was unchanged in P14-HDAC1-cKO^YFP^ cells with the exception of an increased proportion of IFN-γ^+^TNF-α^–^ cells within the severely diminished early Tex^eff-like^ cell population (Fig. S3, A–D). Finally, we directly assessed the cytolytic capacity of HDAC1-deficient Tex cells and performed an in vitro CTL assay with early non-Tex^prog^ cells (i.e., Tex cells other than early Tex^prog^ cells). We adoptively transferred P14-WT or P14-HDAC1-cKO^YFP^ cells into recipient mice, which were infected on the following day, and sorted early non-Tex^prog^ donor cells (i.e., Ly108^−^Tim3^+^ cells) 8 p.i. Subsequently, early non-Tex^prog^ cells were mixed with relevant or irrelevant peptide-pulsed EL-4 target cells and their specific killing assessed (Fig. S3 E). P14-HDAC1-cKO^YFP^ cells lysed target cells as efficiently as P14-WT cells, indicating intact cytolytic capacity of HDAC1-deficient early non-Tex^prog^ cells in vitro (Fig. S3, F and G). Thus, it is likely that the elevated viremia in the mutant mice can be attributed to a significant extent to the reduced numbers of early non-Tex^prog^ cells. However, it remains possible that HDAC1-deficient early non-Tex^prog^ cells (which largely consist of early Tex^nd^ cells) display impaired cytolytic activity in vivo and/or that other alterations in the mutant Tex cells lead to a reduced ability to clear the virus in vivo.

### Lack of HDAC1 does not impair early Tex cell activation, proliferation, or survival

**Figure 5.**
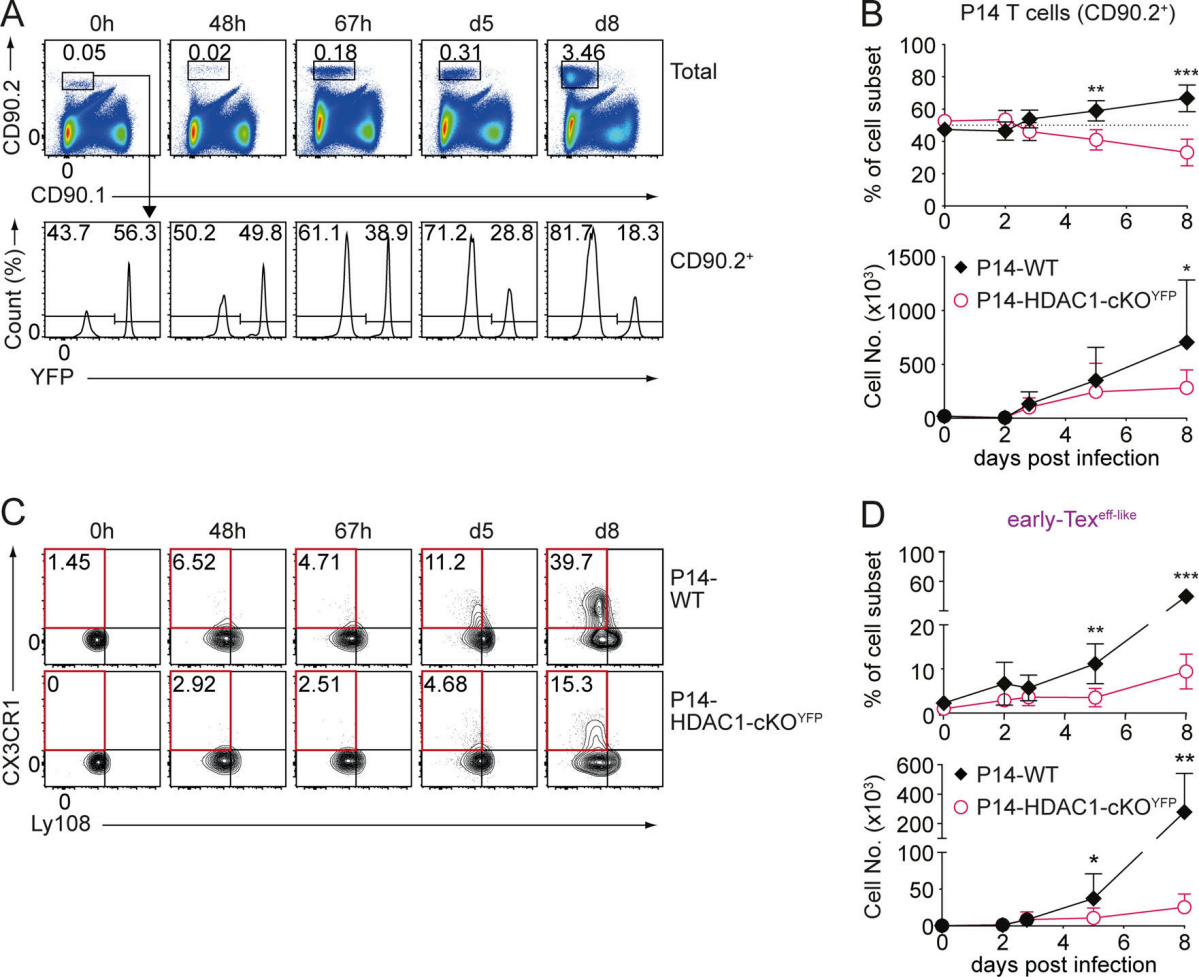
**HDAC1 drives cellular kinetics of virus-specific CD8** ^**+**^ **T cells during onset of infection. (A)** Density plots show percentages of transferred P14 cells (CD90.2^+^) in the spleen of infected mice at various time points of infection (0 h, 48 h, 67 h, day 5 [d5], and d8) (upper panel). P14-WT and P14-HDAC1-cKO^YFP^ cells were distinguished by the expression of YFP (lower panel). **(B)** Summary diagrams show percentages (upper graph) and total numbers (lower graph) of transferred YFP^–^ P14-WT and YFP^+^ P14-HDAC1-cKO^YFP^ cells at various time points p.i. as depicted in A. **(C)** Contour plots show the distribution of P14-WT and P14-HDAC1-cKO^YFP^ Tex subsets at various time points of infection. **(D)** Summary diagrams show percentage (upper graph) and total numbers (lower graph) of early Tex^eff-like^ subsets as depicted in (C, red square). Numbers in the plots (A and C) show the percentage of cells within the indicated regions. Data shown are representative (A and C) or show the summary (B and D) of three to eight mice per time point per genotype from three to five independent experiments. **(B and D)** Data are shown as mean ± SD. *P < 0.05; **P < 0.01; ***P < 0.001; n.s. P ≥ 0.05 (paired two-tailed Student’s *t* test).

Upon the onset of chronic infection, viral-specific CD8^+^ T cells are activated, clonally expand, and differentiate into Tex cells (Chen et al., 2019; Daniel et al., 2022; Utzschneider et al., 2020). To define at which time point upon infection Tex subset composition is altered in the absence of HDAC1, we examined the kinetics of the appearance of CX3CR1^+^ cells at 48 and 67 h as well as day 5 and 8 p.i. and also determined the activation and expansion of early Tex cells. Using the aforementioned adoptive P14 T cell co-transfer model (Fig. 4 A), the ratio of transferred P14-WT to P14-HDAC1-cKO^YFP^ cells up to 67 h p.i. (Fig. 5, A and B) was unchanged. In addition, there was no difference in cell size (as revealed by forward scatter values) or expression levels of the early activation marker CD69 between the two groups at the onset of activation (Fig. S3, H and I). Furthermore, both P14-WT and P14-HDAC1-cKO^YFP^ cells divided at a similar rate based on the “dilution” of division-tracking dye CellTrace Violet (Fig. S3, H and I). These data indicate intact TCR signaling and T cell activation in the absence of HDAC1 at early time points. In contrast, from day 5 p.i. on, P14-HDAC1-cKO^YFP^ cells displayed a relative reduction compared with the P14-WT cells (Fig. 5, A and B). In addition, while the appearance of CX3CR1^+^ cells first became evident at day 5 p.i. in the WT compartment, the proportion of these cells was severely reduced in the absence of HDAC1 (Fig. 5, C and D). However, the deletion of HDAC1 had no impact on the degree of ongoing proliferation as well as the proportion of apoptotic cells (assessed by intracellular Ki67 and active caspase-3 staining, respectively) over the assessed time period of 8 days p.i. (Fig. S3, J and K). Together, these data indicate that HDAC1 does not regulate early activation, proliferation, or survival of cells during early Tex cell differentiation. Rather, HDAC1 might represent a checkpoint that instructs cell fate specification and differentiation toward early Tex^eff-like^ population as early as 5 days p.i.

### HDAC1 is required for the maintenance of the Tex^eff-like^ cell pool

**Figure 6.**
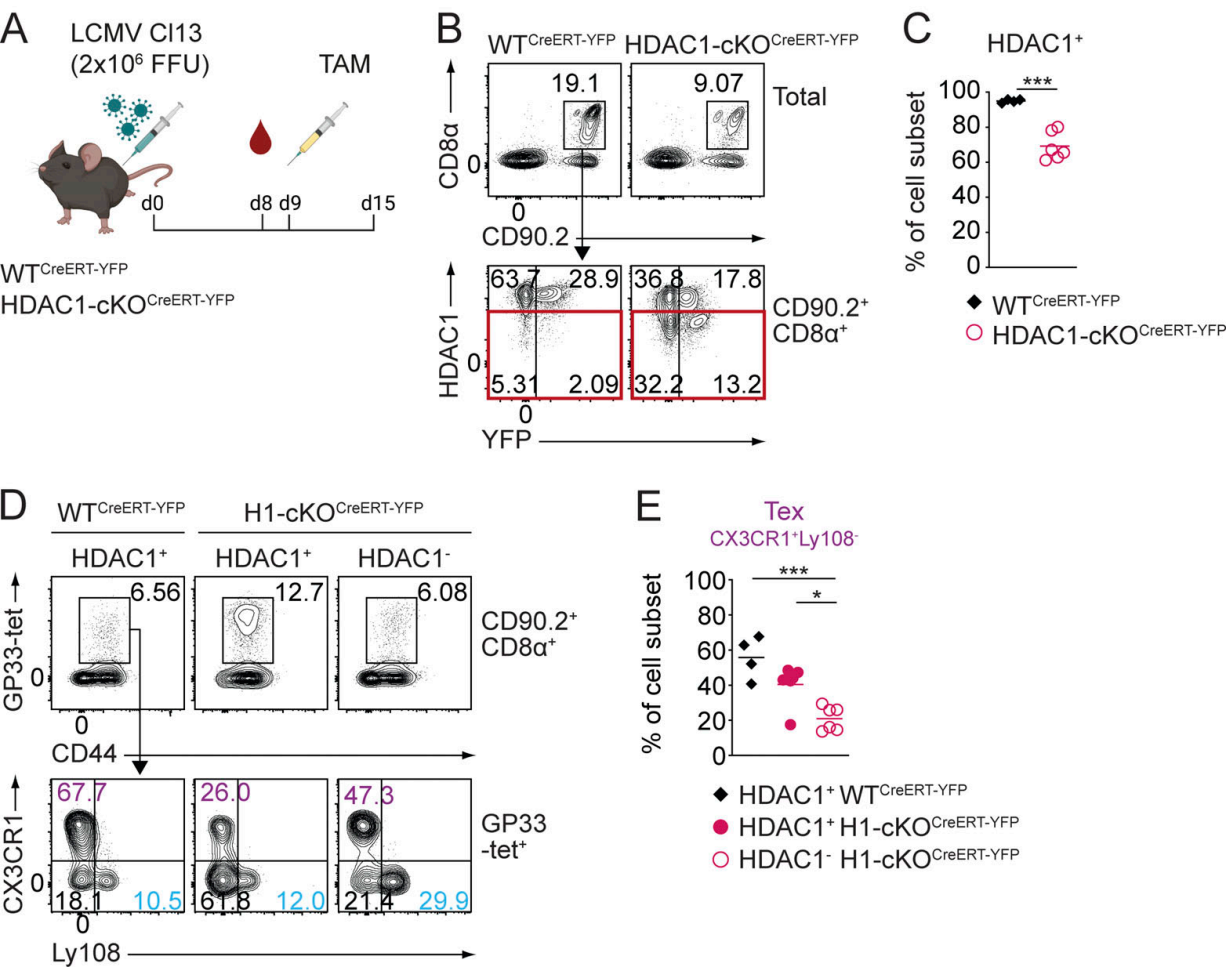
**HDAC1 is required for the maintenance of the Tex** ^**eff-like**^ **cell pool. (A)** Experimental design. WT^CreERT-YFP^ and HDAC1-cKO^CreERT-YFP^ mice were infected with LCMV Cl13. On day 8 (d8) p.i., the composition of Tex subsets was investigated in the blood of infected mice. One day later (d9), mice were injected with 2 mg of tamoxifen (TAM) to induce the deletion of *Hdac1*. Splenocytes were analyzed on d15 p.i. **(B)** Contour plots show the percentage of WT^CreERT-YFP^ and HDAC1-cKO^CreERT-YFP^ CD8^+^ T cells (CD90.2^+^CD8α^+^) in splenocytes (upper panel). CD8^+^ T cells were further characterized as YFP-expressing HDAC1-sufficient (HDAC1^+^) or HDAC1-deficient (HDAC1^−^; red square) cells (lower panel). **(C)** Diagram shows summary of percentage of HDAC1-expressing (HDAC1^+^) CD8^+^ T cells as depicted in B. **(D)** Contour plots show GP33-tet^+^ Tex cells (upper panel) and the distribution of Tex subsets: CX3CR1^–^Ly108^+^ Tex (blue), CX3CR1^+^Ly108^−^ Tex (purple), and CX3CR1^−^Ly108^−^ Tex (black) (lower panel) within HDAC1-sufficient (HDAC1^+^) and HDAC1-deficient (HDAC1^−^) CD8^+^ T cells. **(E)** Summary diagram shows the percentage of CX3XR1^+^Ly108^−^ Tex cell subset as depicted in D. Numbers in plots (B and D) show the percentage of cells within the indicated regions. Data are representative (B and D) or show the summary (C and E) of four to six mice per genotype analyzed in two independent experiments.** (C and E)** Horizontal bars indicate the mean. *P < 0.05; ***P < 0.001; n.s. P ≥ 0.05. **(C)** An unpaired two-tailed Student’s *t* test was used. **(E)** One-way ANOVA followed by Tukey’s multiple comparison test was used.

Our data clearly demonstrate a key role of HDAC1 in the generation of early Tex^eff-like^ cells. Since previous studies have shown that the Tex^eff-like^ cell pool is maintained throughout chronic viral infection (Daniel et al., 2022; Zander et al., 2019), we next examined whether HDAC1 is also essential for the maintenance of Tex^eff-like^ cells. We crossed *Hdac1*^f/f^ mice onto a *Rosa26*-CreERT2 background (Hameyer et al., 2007) to inducibly delete *Hdac1* by tamoxifen administration during the course of chronic viral infection. We also introduced a *Rosa26*-STOP-EYFP reporter allele to monitor Cre activity and thus cells that have deleted *Hdac1*, leading to the generation of *Hdac1*^f/f^, *Rosa26*-CreERT2/STOP-YFP, and *Hdac1*^+/+^, *Rosa26*-CreERT2/STOP-YFP mice (hereafter referred to as WT^CreERT-YFP^ and HDAC1-cKO^CreERT-YFP^ mice, respectively). In the absence of tamoxifen, YFP expression was not induced, and the distribution of circulating Tex cells was normal 8 days p.i., indicating that the genetic system is tightly controlled as expected (Fig. S3, L and M). Subsequently, mice were treated with tamoxifen on day 9 p.i., and the distribution of splenic Tex cells was analyzed 6 days later (i.e., 15 days p.i.) (Fig. 6 A). After tamoxifen injection, YFP expression was induced in ∼30% of CD8^+^ T cells. However, intracellular anti-HDAC1 staining revealed that YFP expression did not correlate with the deletion of *Hdac1* and hence loss of HDAC1 expression, which was detected in ∼40% of CD8^+^ T cells (Fig. 6, B and C). Therefore, we eventually identified HDAC1-deficient cells in HDAC1-cKO^CreERT-YFP^ mice by “gating” on the HDAC1-negative (HDAC1^−^) population based on the intracellular anti-HDAC1 staining (i.e., cells in which HDAC1 expression was not detected due to deletion of the *Hdac1* gene) (Fig. 6 B; red square). Strikingly, the analysis of the distribution of Tex cells revealed a reduction in the proportion of the CX3CR1^+^ cells within the HDAC1^−^ population in HDAC1-cKO^CreERT-YFP^ mice compared with WT^CreERT-YFP^ mice as well as to the HDAC1^+^ population of HDAC1-cKO^CreERT-YFP^ mice (Fig. 6, D and E). Taken together, our results demonstrate a CD8^+^ T cell–intrinsic key role for HDAC1 not only for the generation but also for the maintenance of the Tex^eff-like^ cell population during chronic viral infection.

### scRNA-seq reveals distinct cell clusters in WT and HDAC1-deficient early Tex cells

**Figure 7.**
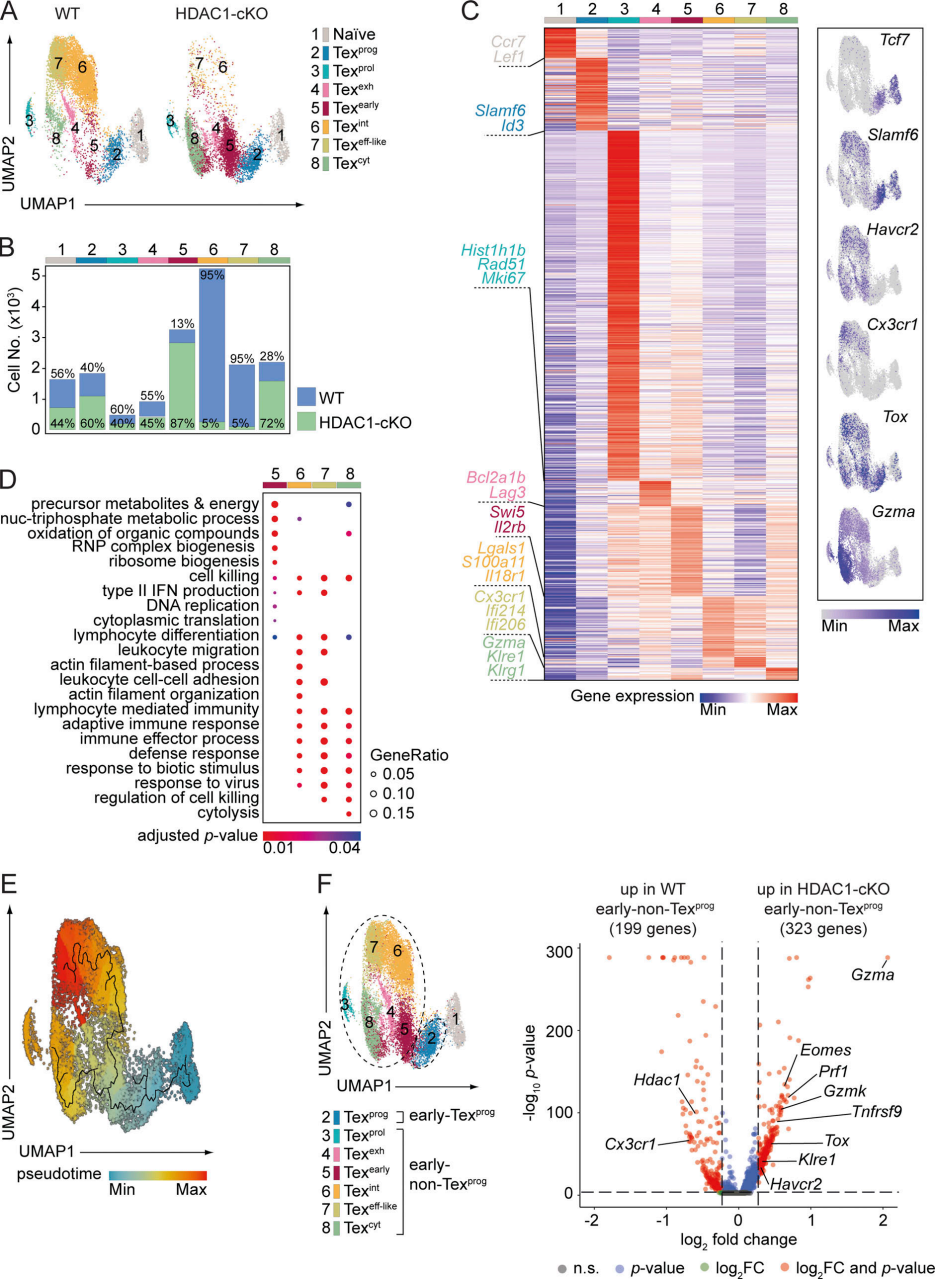
**scRNA-seq reveals transcriptionally distinct cell clusters between LCMV-specific WT and HDAC1-deficient CD8** ^**+**^ **T cells. (A)** UMAP plots display clusters identified from a pool of naïve and virus-specific (GP33-tet^+^) WT and HDAC1-cKO T cells from uninfected and from day 8 LCMV Cl13–infected mice, respectively. (C1) T^naive^; (C2) Tex^prog^: progenitor; (C3) Tex^prol^: proliferating; (C4) Tex^exh^: exhausted; (C5) Tex^early^: early; (C6) Tex^int^: intermediate; (C7) Tex^eff-like^: effector-like; (C8) Tex^cyt^: cytotoxic. **(B)** Stacked bar graph shows the number of WT or HDAC1-cKO CD8^+^ T cells within each cluster as defined in A. Numbers in the bars indicate the frequency of WT or HDAC1-cKO CD8^+^ T cells within a particular cluster. **(C)** Left: heatmap showing the average expression of all genes unique for each cluster as defined by FindAllMarkers() from the Seurat package using default parameters. Selected genes representative of each cluster are shown. Right: feature plots of selected subset-specific marker genes, indicating the expression level in each cell. Contrast was improved by using minimum and/or maximum cutoffs, and positive cells were plotted on top. **(D)** Bubble plot showing significantly enriched (adjusted P value <0.05) GO terms (biological processes) in the WT- (C6 and C7) and HDAC1-cKO– (C5 and C8) dominant clusters obtained from clusterProfiler. Top 200 markers of each cluster were used for enrichment analysis. Top five enriched pathways from each cluster were collapsed to parent terms using Revigo and then visualized. Bubble size corresponds to the ratio of marker genes overlapping with the given pathway (Gene Ratio), and bubble color corresponds to the adjusted P value. **(E)** Single-cell trajectory of Tex cells according to the cell subsets present at day 8 p.i. Color scheme proceeds from dark blue to dark red as cells proceed through their trajectory (pseudotime), which predicts a differentiation path starting from the naïve T cell cluster, passing through the Tex^prog^ and Tex^early^ cell clusters, and then bifurcating into either the Tex^int^ and Tex^eff-like^ clusters or Tex^cyt^ cluster. **(F)** Left: UMAP shows the defined early Tex^prog^ cell cluster (C2) and the early non-Tex^prog^ cell cluster (C3–8). Right: volcano plot showing differentially expressed genes between WT and HDAC1-cKO early non-Tex^prog^ cells. Horizontal dashed line at P value of 0.05, vertical dashed lines at log_2_FC of −0.25 and 0.25, FC: fold change, n.s.: not significant. Numbers on plots represent the cluster number as assigned by scRNA-seq analysis (A and F) or the frequencies within the indicated regions (B). UMAP, uniform manifold approximation and projection.

**Figure S4.**
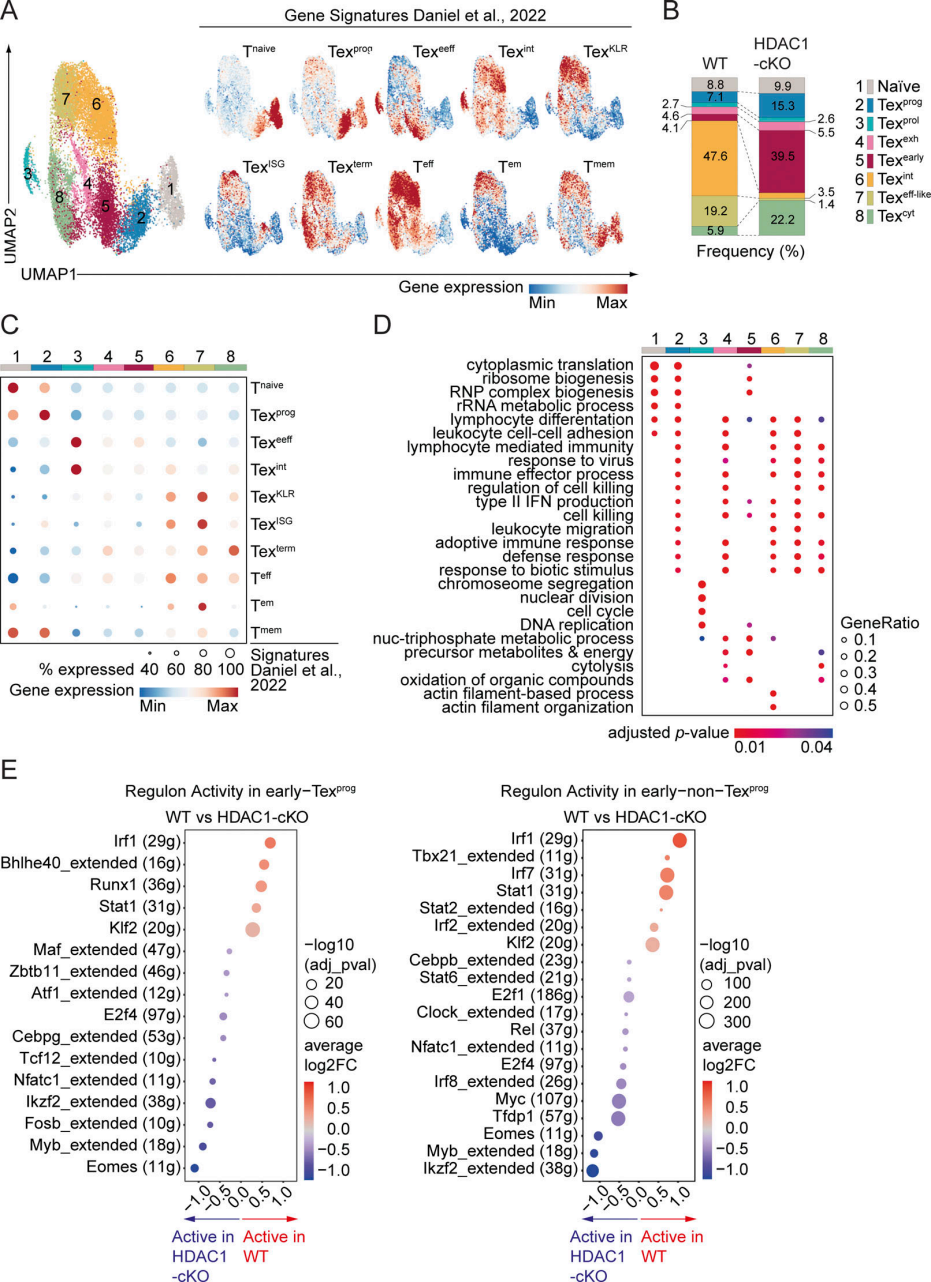
**scRNA-seq reveals transcriptionally distinct cell clusters between LCMV-specific WT and HDAC1-deficient CD8** ^**+**^ **T cells. (A)** UMAPs show clustering of naïve and virus-specific WT and HDAC1-cKO CD8^+^ T cells from noninfected or day 8 LCMV Cl13–infected mice (left). Feature plots (right) show the expression of gene signatures from a published data set (Daniel et al., 2022) projected onto our scRNA-seq data. Contrast was improved by using minimum and/or maximum cutoffs, and positive cells were plotted on top. **(B)** Stacked bar graph depicts the frequency of each cluster within the total of WT and HDAC1-cKO CD8^+^ T cells. **(C)** Bubble plot shows comparison of gene signatures of WT and HDAC1-cKO clusters to a published dataset (Daniel et al., 2022). Bubble size corresponds to the percentage of genes overlapping with the published gene sets, and bubble color corresponds to the average expression of overlapping genes. **(D)** Bubble plot shows significantly (adjusted P value <0.05) enriched GO terms (biological processes) in all clusters obtained from clusterProfiler. Top 200 markers of each cluster were used for enrichment analysis. Top five enriched pathways from each cluster were collapsed to parent terms using Revigo and then visualized. Bubble size corresponds to the ratio of marker genes overlapping with the given pathway (Gene Ratio), and bubble color corresponds to the adjusted P value. **(E)** Bubble plots indicate regulon activities increased in WT (red) or HDAC1-cKO (blue) early Tex^prog^ (left panel) and early non-Tex^prog^ (right panel) cells, as revealed by the SCENIC analysis. Bubble size corresponds to the adjusted P value, and bubble color corresponds to the average fold changes. Numbers in parentheses indicate the number of downstream target genes co-expressed with each TF. UMAP, uniform manifold approximation and projection.

To gain further insight into the impact of HDAC1 deletion on Tex subset diversity at early stages during chronic infection, we performed scRNA-seq of naïve and LCMV-specific (GP33-tet^+^) CD8^+^ T cells isolated from WT and HDAC1-cKO mice, encompassing uninfected and day 8 infected conditions, respectively. Uniform manifold approximation and projection followed by Seurat-based clustering led to the identification of eight clusters (Fig. 7, A and B; and Table S1), which were subsequently annotated based on their unique marker genes (Fig. 7 C) as well as their similarity to published signature genes of CD8^+^ T cell subsets upon acute or chronic LCMV infection (Fig. S4, A–C) (Daniel et al., 2022). We found two canonical clusters corresponding to naïve (cluster C1; T^Naïve^) and early Tex^prog^ cells (C2; Tex^prog^). Furthermore, two other relatively small clusters were identified that highly expressed genes related to either cell proliferation (e.g., *Hist1h1b* and *Mki67*) (C3; Tex^prol^) or terminal exhaustion (e.g., *Bcl2a1d* and *Lag3*) (C4; Tex^exh^) (Fig. 7, A–C and Fig. S4, A–C). The majority of cells (∼77% of WT and 67% of HDAC1-cKO cells) were grouped into four other clusters (C5–C8) (Fig. S4 B). Cluster C5 (Tex^early^) was enriched for a gene signature of Tex^eeff^ cells (early effector exhausted cells where the Tex program is initiated [Daniel et al., 2022]) (Fig. S4, A and C). Cluster C6 (Tex^int^) expressed the *Cx3cr1* gene and was enriched for a gene signature of Tex^int^ cells (intermediate exhausted cells harboring potential to become both Tex^term^ and Tex^eff-like^ cells [Daniel et al., 2022]) (Fig. 7 C; and Fig. S4, A and C). Cluster C7 (Tex^eff-like^) expressed the *Cx3cr1* gene at a higher level than the C6 cluster and was enriched for a gene signature of Tex^KLR^ cells (KLR family member protein-expressing Tex cells, a major subpopulation of Tex^eff-like^ cells [Daniel et al., 2022]) (Fig. 7 C; and Fig. S4, A and C). Lastly, cluster C8 (Tex^cyt^) highly expressed genes encoding cytolytic proteins but also displaying the highest enrichment score of a Tex^term^ gene signature (Daniel et al., 2022) (Fig. 7 C; and Fig. S4, A and C). Notably, while the C1–C4 clusters (T^Naïve^, Tex^prog^, Tex^prol^, and Tex^exh^) were almost equally composed of WT and HDAC1-cKO cells, cluster C6 (Tex^int^) and cluster C7 (Tex^eff-like^) contained mostly WT cells (Fig. 7, A and B; and Fig. S4 B). In contrast to the C6 and C7 clusters, the vast majority of the C5 (Tex^early^) and C8 (Tex^cyt^) clusters consisted of HDAC1-deficient cells (Fig. 7, A and B; and Fig. S4 B). A gene ontology (GO) analysis of clusters C5–C8 revealed that several biological processes (e.g., ribonucleoprotein complex biogenesis) were uniquely overrepresented in the HDAC1-cKO–dominant C5 cluster (Tex^early^) (Fig. 7 D and Fig. S4 D). In addition, while WT-dominant clusters C6 and C7 shared almost identical enriched terms for GO biological processes, some of these processes (e.g., “leukocyte migration”) were not enriched in the HDAC1-cKO–dominant C5 and C8 (Fig. 7 D and Fig. S4 D). Instead, cluster C8 shared certain processes with C5 (e.g., “oxidation of organic compounds”) (Fig. 7 D and Fig. S4 D). This analysis suggests that the lack of HDAC1 leads to the emergence of transcriptionally distinct subsets within the early non-Tex^prog^ population (i.e., Tex cells other than early Tex^prog^ cells), in contrast to its minor impact on the generation of early Tex^prog^ cells. The specific role of HDAC1 for the formation of early non-Tex^prog^ cells was further supported by a trajectory inference analysis (Cao et al., 2019), which predicted that Tex^prog^ cells bifurcate into Tex^eff-like^ and Tex^cyt^ clusters, with a major branch point at the Tex^early^ cell cluster (Fig. 7 E). A comparison of gene expression between WT and HDAC1-cKO early non-Tex^prog^ cells showed that the lack of HDAC1 led to increased expression of genes associated with cytolytic function (e.g., *Gzma*, *Gzmk*, *Prf1*, and *Klre1*) and exhaustion (e.g., *Havcr2*, *Tox*, *Tnfrsf9*, and *Eomes*), concurrent with downmodulation of *Cx3cr1* gene expression (Fig. 7 F). This is in line with our immunophenotyping of early Tex cells (Fig. 3, A and B), such as the simultaneous up- and downmodulation of KLRG1 and CX3CR1, respectively, in HDAC1-cKO early non-Tex^prog^ cells, whose expression levels are positively correlated during Tex cell differentiation in a HDAC1-sufficient setting (Beltra et al., 2020; Zander et al., 2019).

Finally, to investigate the impact of HDAC1 deletion on gene regulatory networks in early Tex^prog^ and early non-Tex^prog^ cells, we compared the activity of regulons (i.e., co-expression modules consisting of a TF and its putative direct targets within the same cell) between WT and HDAC1-deficient cells (Fig. S4 E). This analysis identified distinct sets of TFs whose regulon activities were elevated either in WT or in HDAC1-deficient Tex cells. Among these TFs, T-bet activity (encoded by the *Tbx21* gene), which is essential for the generation of Tex^eff-like^ cells (Beltra et al., 2020; Raju et al., 2021; Zander et al., 2019), was upregulated in WT early non-Tex^prog^ cells. In addition, the regulon activity of Eomes was increased in HDAC1-cKO early Tex^prog^ and early non-Tex^prog^ cells. Of note, relative expression levels of Eomes and T-bet are linked with the progression of terminal exhaustion (Beltra et al., 2020; Zander et al., 2019). Since we observed increased (or a trend toward an increase) expression levels of T-bet and Eomes proteins in WT and HDAC1-cKO early Tex cells, respectively (Fig. 3, A and B; and Fig. 4, G and H), it is likely that HDAC1 controls early Tex^eff-like^ cell differentiation, at least in part, by regulating the expression and/or function of these two TFs. Thus, the deletion of HDAC1 resulted in specific alterations in transcriptional networks controlling early Tex cell differentiation and the enlargement of two Tex clusters with early exhaustion (C5) and exhaustion/cytolysis (C8) signatures.

### HDAC1 alters the chromatin landscape at early Tex^eff-like^ signature gene loci

**Figure 8.**
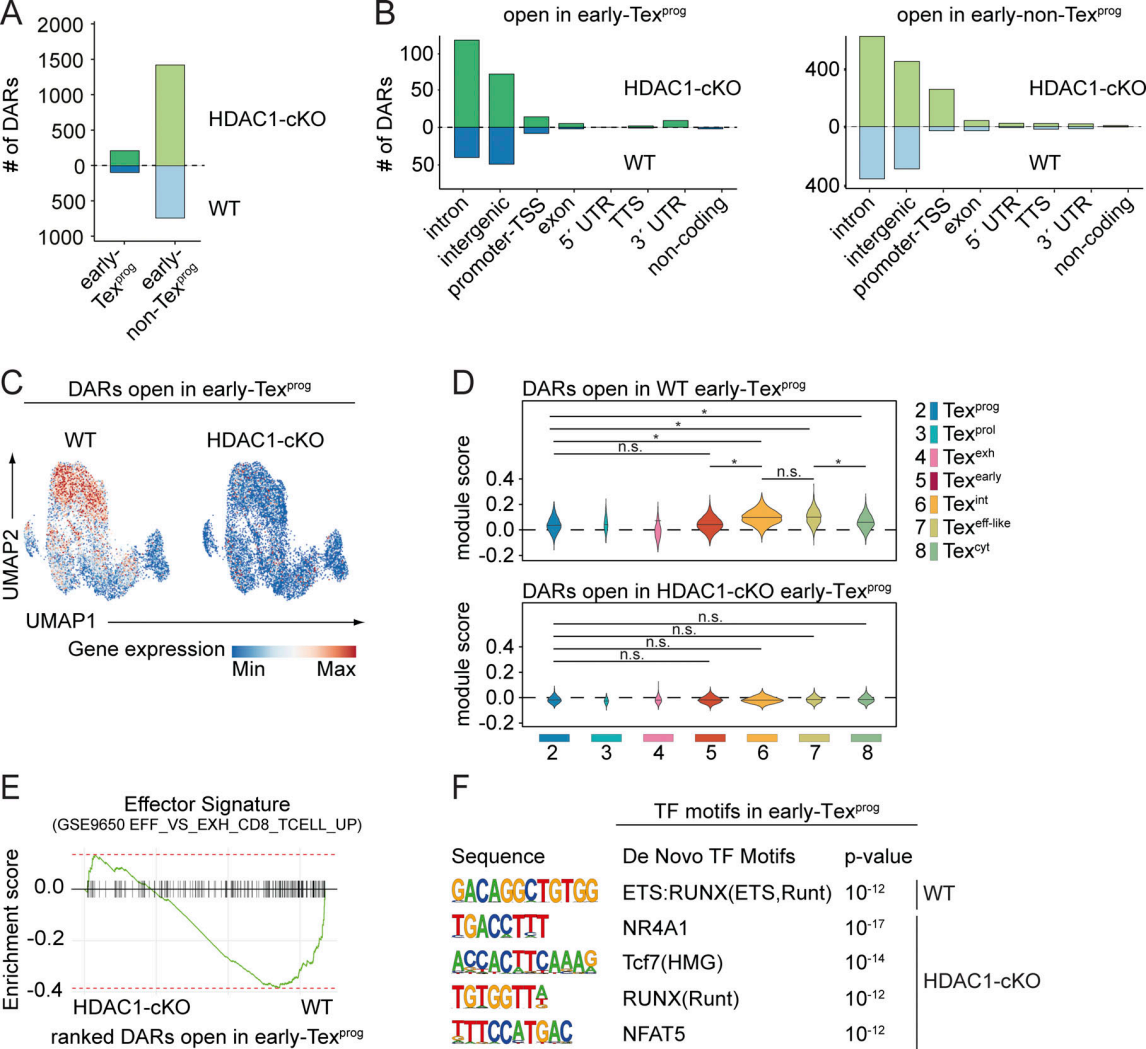
**HDAC1 alters the chromatin landscape at signature gene loci associated with early Tex** ^**eff-like**^ **cells. (A)** Diagram indicates the number of DARs (adjusted P value <0.05) between WT and HDAC1-cKO early Tex^prog^ and early non-Tex^prog^. **(B)** Number of DARs in early Tex^prog^ (left) and early non-Tex^prog^ (right) split by their detailed genomic annotation obtained by HOMER. TSS: transcription start site (−1 kb to +100 bp around TSS), TTS: transcription termination site (−100 bp to +1 kb around TTS). **(C)** UMAP plot depicting the expression of genes (as revealed by scRNA-seq) associated with open DARs in either WT (left) or HDAC1-cKO (right) early Tex^prog^ from ATAC-seq. **(D)** Violin plots depict the average expression of genes associated with open DARs in WT early Tex^prog^ cells (upper panel) or HDAC1-cKO early Tex^prog^ cells (lower panel), calculated as module scores for the clusters C2–C8 as defined by scRNA-seq. ANOVA followed by Tukey’s test was performed, * adjusted P value <0.05, n.s. not significant. **(E)** Gene set enrichment analysis shows Effector Signature (from ImmuneSigDB) of ranked genes associated with DARs open in either WT or HDAC1-cKO early Tex^prog^ cells. The barcodes indicate the location of the genes associated with DARs in the ranked list that intersect with the respective signature. **(F)** TF motifs within DARs open in either WT or HDCA1-cKO early Tex^prog^ cells as determined by de novo motif analysis using HOMER (P values <10^−11^ were considered true positive). UMAP, uniform manifold approximation and projection.

**Figure S5.**
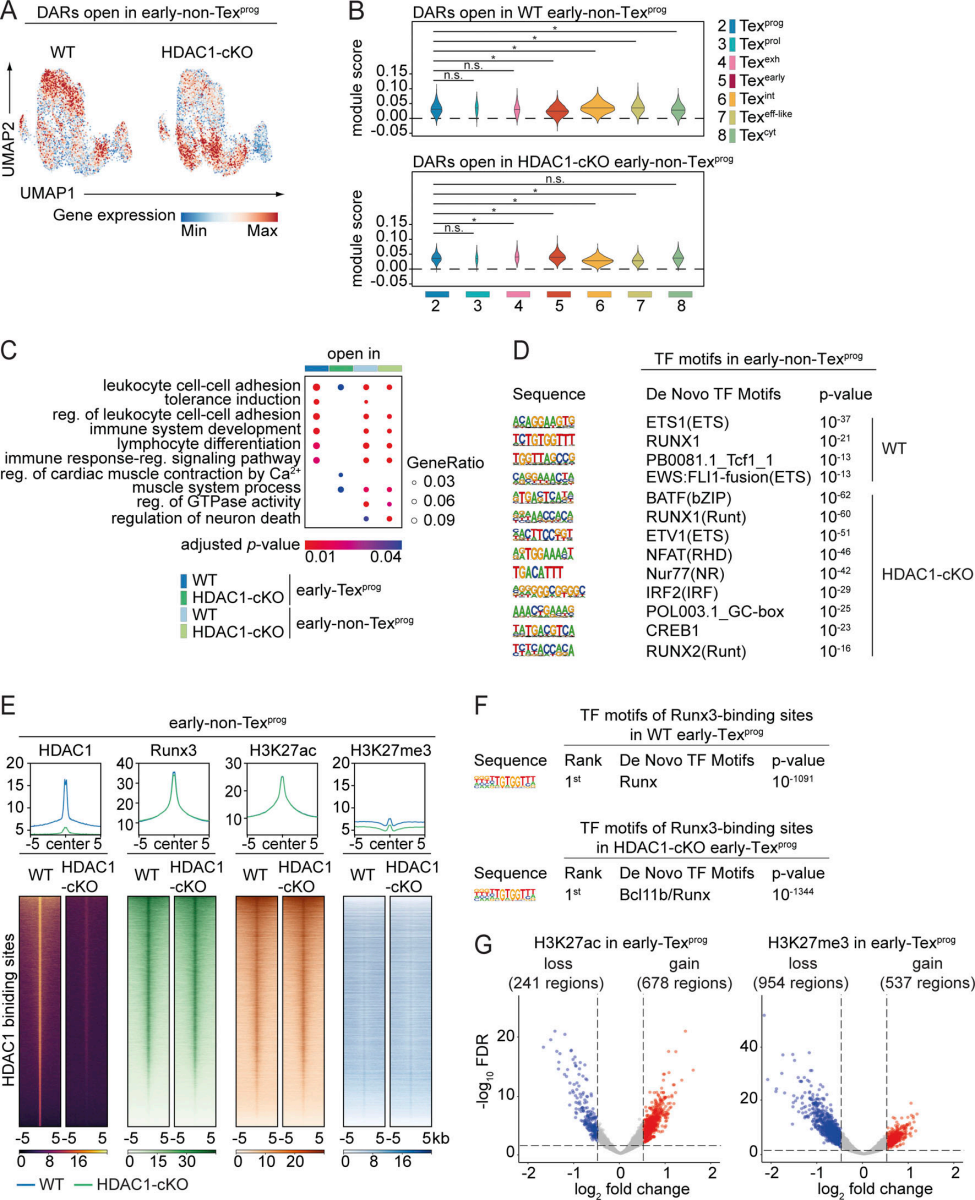
**HDAC1 alters the chromatin landscape required for the maintenance of the Tex** ^**eff-like**^ **cell pool. (A)** Expression of gene transcripts in scRNA-seq of genes associated with open DARs in early non-Tex^prog^ from ATAC-seq. **(B)** Violin plots depict the average expression of genes associated with open DARs in WT early nonTex^prog^ cells (upper panel) or HDAC1-cKO early non-Tex^prog^ cells (lower panel), calculated as module scores for clusters C2–C8 as defined by scRNA-seq. ANOVA followed by Tukey’s test was performed; * adjusted P value <0.05; n.s. not significant. **(C)** Bubble plot showing significantly enriched (adjusted P value <0.05) GO terms (biological processes) in genes associated with open DARs of WT and HDAC1-cKO early Tex^prog^ and early non-Tex^prog^ cells. Bubble size corresponds to the ratio of genes overlapping with the given pathway (Gene Ratio), and bubble color corresponds to the adjusted P value. **(D)** TF motifs accessible in WT and HDAC1-cKO early non-Tex^prog^ cells as determined by de novo motif analysis using HOMER (P values <10^−11^ were considered true positive). **(E)** Heatmaps and profile plots indicating genome-wide HDAC1 and RUNX3 binding, as well as H3K27 acetylation (ac) and H3K27 tri-methylation (me3) marks, in WT and HDAC1-cKO early non-Tex^prog^ cells as detected by CUT&RUN. Peaks for RUNX3, H3K27ac, and H3K27me3 marks are centered around HDAC1-binding site ± 5 kb. Blue lines indicate WT values; green lines indicate HDAC1-cKO values. **(F)** Enriched TF motifs ranked first within RUNX3-binding regions detected by CUT&RUN in WT and HDAC1-cKO early Tex^prog^ cells as determined by de novo motif analysis using HOMER. **(G)** Volcano plots showing gain and loss of H3K27ac (left panel) and H3K27me3 (right panel) upon HDAC1 deletion detected by CUT&RUN in early Tex^prog^. Fold changes >0.05 and FDR < 0.05 were considered significant. Red dots represent regions, which gain acetylation or methylation in HDAC1-cKO cells, and blue dots represent regions, which lose acetylation or methylation in HDAC1-cKO cells. Log_2_ fold change and −log_10_ FDR are plotted on the x and y axis, respectively.

Transcriptional changes during Tex subset differentiation are associated with dynamic shifts in chromatin accessibility (Beltra et al., 2020; Chen et al., 2021a). Given that our data show no alteration in the activation, proliferation and survival of early Tex cells (Fig. S3, H–K) as well as the intact generation of early Tex^prog^ cells lacking HDAC1 (Fig. 7, A and B), it is conceivable that HDAC1 controls the transition of early Tex^prog^ cells into early Tex^eff-like^ cells, at least in part, by mediating changes in the chromatin landscape. To examine this hypothesis, we conducted bulk ATAC-seq of P14-WT and P14-HDAC1-cKO early Tex^prog^ (Ly108^+^Tim3^−^) as well as early non-Tex^prog^ (Ly108^−^Tim3^+^) cells 8 days p.i. A comparison between WT and HDAC1-cKO cells revealed 319 and 2,179 differentially accessible regions (DARs) in early Tex^prog^ and early non-Tex^prog^ cells, respectively (Fig. 8, A and B; and Table S2). The vast majority of the DARs were located within introns, intergenic regions, or at promoters in both populations. Lack of HDAC1 generally led to increased numbers of “open” chromatin regions in both populations, in line with the known function of HDACs in promoting a “closed” chromatin structure (Fig. 8, A and B). However, 102 (at 97 gene loci) and 217 DARs (at 201 gene loci) were more open in WT early Tex^prog^ and early non-Tex^prog^ cells. We next explored whether there is a correlation between changes in chromatin accessibility and gene expression by integrating the data obtained from bulk ATAC-seq and scRNA-seq. We defined the gene sets harboring DARs either in WT or HDAC1-cKO cells and determined the overall average expression levels (i.e., the module scores) of these gene sets at single-cell level (Fig. 8 C and Fig. S5 A). Moreover, we calculated the average module scores within the individual clusters (i.e., C2–C8) and thereby examined whether DARs-associated genes are preferentially expressed in certain cluster(s) (Fig. 8 D and Fig. S5 B). Notably, this analysis revealed that the 97 genes with 102 DARs more accessible in WT early Tex^prog^ cells are highly expressed in WT-dominant cluster C6 (Tex^int^) and C7 (Tex^eff-like^), indicating a role for HDAC1 in priming the expression of early Tex^eff-like^ cell–associated genes by “opening” their loci in early Tex^prog^ cells (Fig. 8, C and D). In contrast, for the genes associated with DARs more accessible in HDAC1-deficient early Tex^prog^ cells, there was no enrichment to a particular cluster (Fig. 8, C and D). Overrepresentation analysis of GO terms showed that genes associated with DARs more accessible in WT early Tex^prog^ cells are also enriched in GO terms such as “leukocyte cell–cell adhesion” and “lymphocyte differentiation” (Fig. S5 C), similar to the single-cell transcriptome of WT-dominant clusters C6 and C7 (Fig. 7 D). Moreover, gene set enrichment analysis of DAR-associated genes revealed the overrepresentation of an effector T cell signature (i.e., a signature of total CD8^+^ T cells on day 8 p.i. with acute LCMV Armstrong) (Wherry et al., 2007) in WT cells in comparison to the HDAC1-cKO cells (Fig. 8 E). These results further support a role for HDAC1 in priming an early Tex^eff-like^ program. Of note, genes more accessible in WT or HDAC1-cKO early non-Tex^prog^ cells were highly expressed in WT- or HDAC1-cKO–dominant clusters (i.e., C6/C7 or C5/C8), respectively, indicating a correlation of accessibility and gene expression in early non-Tex^prog^ cells (Fig. S5, A and B). Finally, an enrichment analysis of TF-binding motifs revealed shared as well as unique TF-binding motifs enriched in DARs of WT and/or HDAC1-cKO cells, both in early Tex^prog^ cells as well as in the early non-Tex^prog^ cell population (Fig. 8 F and Fig. S5 D). This suggests that the same TFs, in particular members of the Runx family, might be recruited to different gene loci and that a different set of TFs might be active, dependent on the presence or absence of HDAC1. Together, the integration of our ATAC-seq analysis with our scRNA-seq data indicates that HDAC1 is essential for an open chromatin state at effector-like signature gene loci in early Tex^prog^ cells.

### HDAC1 primes an open chromatin state at early Tex^eff-like^ signature gene loci in early Tex^prog^ cells

**Figure 9.**
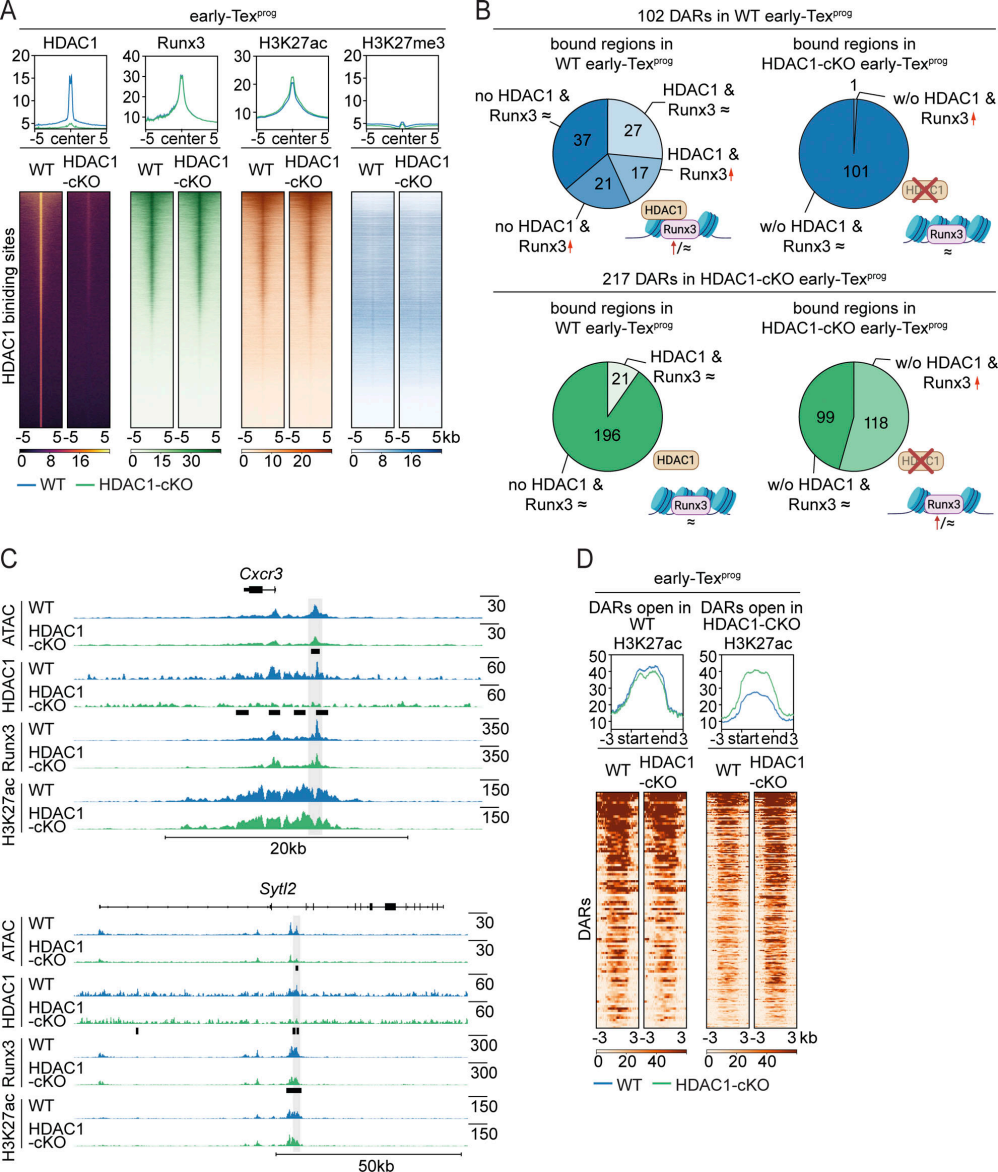
**HDAC1 binding primes the opening of effector-like signature gene loci in early Tex** ^**prog**^ **cells. (A)** Heatmaps and profile plots visualizing changes in RUNX3 binding, H3K27 acetylation (ac), and tri-methylation (me3) intensity in WT and in HDAC1-deficient early Tex^prog^ cells in all sites bound by HDAC1 in WT cells. Peaks are centered around HDAC1-binding sites ± 5.0 kb. Blue lines indicate WT values; green lines indicate HDAC1-cKO values. **(B)** Pie charts showing the numbers of HDAC1 and RUNX3-binding sites detected by CUT&RUN, which overlap with DARs of WT and HDAC1-cKO early Tex^prog^ cells detected by ATAC-seq. Upper pie charts: HDAC1 and RUNX3 binding in WT (left) and HDAC1-cKO (right) early Tex^prog^ cells to 102 DARs more open in WT cells. Lower pie chart: HDAC1 and RUNX3 binding in WT (left) and HDAC1-cKO (right) early Tex^prog^ cells to 217 DARs more open in HDAC1-cKO cells. Runx3 ≈: recruitment to the DARs is unchanged between genotypes; Runx3 ↑: recruitment is increased in the corresponding genotype compared with the other. **(C)** Changes on chromatin accessibility, HDAC1, and RUNX3 binding, as well as H3K27ac levels in the absence of HDAC1 at the *Cxcr3* and *Sytl2* gene loci. Tracks show normalized coverage of each factor, and horizontal bars indicate DARs for ATAC-seq or differential binding of HDAC1 and Runx3 for CUT&RUN analysis. Of note, H3K27ac level is unchanged between the two genotypes at these two loci. RefSeq gene annotations are indicated on top. **(D)** H3K27ac changes in DARs in early Tex^prog^ cells from ATAC-seq upon HDAC1 deletion. Heatmaps and profile plots show the respective consensus regions from ATAC-seq ± 3.0 kb. Left panel: H3K27ac intensities in DARs more open in WT cells. Right panel: H3K27ac intensities in DARs more open in HDAC1-cKO cells.

To investigate whether the appearance of open ATAC-seq regions correlates with the binding of HDAC1 and whether HDAC1 regulates histone acetylation levels at these gene loci, we performed CUT&RUN for HDAC1, H3K27ac, and H3K27me3 in WT and HDAC1-cKO early Tex cells. Moreover, since DARs that are more open in WT cells showed an enrichment of Runx-binding motifs, we also performed CUT&RUN for Runx3, which plays an essential role in the initiation of CD8^+^ T cell effector programs in acute (Shan et al., 2017; Wang et al., 2018) and chronic viral infections (Chen et al., 2021b; Shan et al., 2021). On a genome-wide level, a total of 42,013 HDAC1-binding sites at 16,276 gene loci in WT early Tex^prog^ cells and 44,414 HDAC1-binding sites at 15,404 gene loci in WT early non-Tex^prog^ cells were identified (Fig. 9 A and Fig. S5 E). The heatmaps of H3K27ac and H3K27me3 modifications revealed an inverse correlation, in agreement with the opposite functions of these two histone marks (Millán-Zambrano et al., 2022; Zhao and Garcia, 2015). We also observed a strong enrichment for Runx-binding motifs in the Runx3 CUT&RUN (Fig. 9 A; and Fig. S5, E and F). The deletion of HDAC1 had a minor impact on genome-wide H3K27ac sites in early Tex^prog^ cells, with 678 upregulated sites (on 571 gene loci) and 241 downregulated sites (on 189 gene loci) of a total of 11,866 sites (Fig. S5 G). 510 of the 571 gene loci were bound by HDAC1, suggesting a direct control of these H3K27ac sites by HDAC1. Although this is in line with the well-known functions of HDACs (i.e., removal of acetylation marks at histones), our data indicate that HDAC1 is not the main epigenetic eraser for H3K27ac marks in early Tex^prog^ cells. With respect to H3K27me3 modifications, deletion of HDAC1 also had only a minor impact with a down- and upregulation of 954 and 537 sites (of a total 18,979 sites), respectively (Fig. S5 G).

**Figure 10.**
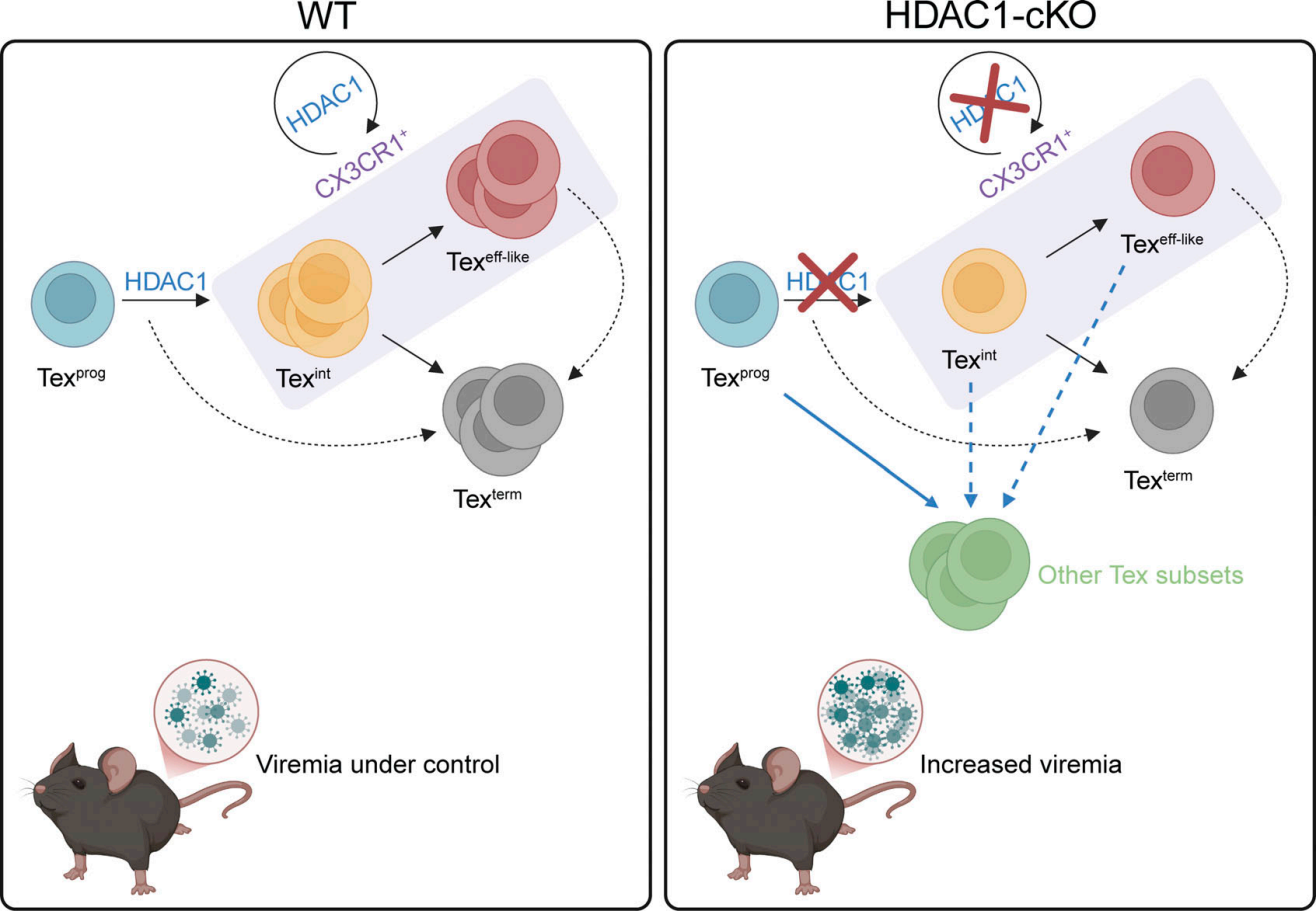
**Working model of how HDAC1 drives Tex subset differentiation.** Left Panel: In WT CD8^+^ T cells, HDAC1 facilitates the proper transition of Tex^prog^ into Tex^int^ cells. The latter cells further differentiate into either Tex^eff-like^ or Tex^term^ (the bifurcation model—straight lines). Although Tex^int^ and Tex^eff-like^ are transcriptionally distinct, they are not well-distinguishable by flow cytometry, as both express the surface marker CX3CR1 (marked by purple rectangle). We have also shown that HDAC1 in T cells is essential for the maintenance of the CX3CR1^+^ Tex subset (including both Tex^int^ and Tex^eff-like^ cells). However, we cannot formally exclude that Tex^term^ subsets originate directly from Tex^prog^ cells or from Tex^eff-like^ cells (dotted lines). Proper Tex subset differentiation in the presence of HDAC1 keeps viremia under control. Right Panel: A T cell–specific deletion of HDAC1 impairs Tex subset differentiation into CX3CR1^+^ Tex^int^ and Tex^eff-like^ cells, resulting in the expansion of other Tex subsets (including Tex^early^ and Tex^cyt^ clusters). It is conceivable that the differentiation block toward CX3CR1^+^ Tex cells promotes the differentiation of these other Tex subsets from either Tex^prog^, Tex^int^, or Tex^eff-like^ cells (blue lines). Decreased numbers and/or impaired maintenance of the CX3CR1 population in the absence of HDAC1 also results in ineffective viral clearance and thus increased viremia.

Next, we focused on the 102 ATAC-seq regions that are more open in WT early Tex^prog^ cells at the 97 gene loci that are later expressed in WT early non-Tex^prog^ cells (i.e., predominantly consisting of Tex^int^ and Tex^eff-like^ clusters). We observed HDAC1 binding at 67 of these 97 gene loci, indicating that ∼69% of these loci are controlled by HDAC1. The majority of HDAC1-binding sites (44 out of 67) overlapped with the open ATAC-seq regions (Fig. 9, B and C), showing an unexpected correlation of HDAC1 binding with open chromatin. On the contrary, from the 217 regions at 201 gene loci that are more open in HDAC1-cKO early Tex^prog^ cells compared with WT cells, only 75 genes (∼37%) showed HDAC1 binding. From the 75 binding sites, only 21 overlapped with open ATAC-seq regions. This indicates that the frequency of HDAC1 binding to gene loci that are more open in WT early Tex^prog^ cells than in HDAC1-cKO Tex^prog^ cells is much higher compared with the gene loci that are more open when HDAC1 is deleted. Furthermore, the appearance of the open 102 ATAC-seq regions in WT early Tex^prog^ did not lead to an overall increase in H3K27ac levels at these regions, while H3K27ac marks were increased at the 217 ATAC-seq regions that are open in HDAC1-cKO early Tex^prog^ cells (Fig. 9 D). Finally, we detected increased recruitment of Runx3 in WT early Tex^prog^ cells (compared with the corresponding HDAC1-cKO subset) at 17 of the 44 open chromatin regions bound by HDAC1 (Fig. 9, B and C; as exemplified by the *Sytl2* gene). This suggests that approximately half of the effector-like genes, whose expression is primed by direct HDAC1 recruitment, might be co-regulated by HDAC1 and Runx3. Together, these data suggest an unexpected mode of action for HDAC1 and that HDAC1, in part together with Runx3, primes effector gene loci in Tex^prog^ cells (Fig. 10 A).

## Discussion

Here, we discovered that HDAC1 is an integral part of the differentiation program endowing Tex cells with effector-like characteristics during chronic viral infection. The molecular mechanisms that direct Tex^prog^ into Tex^eff-like^ or Tex^term^ cell subsets remain largely unknown (Kanev and Zehn, 2021; Zander and Cui, 2023). Based on our data showing a reduction of Tex^eff-like^ cells in the absence of HDAC1, HDAC1 might promote early Tex^prog^ toward early Tex^eff-like^ differentiation or suppress alternative early Tex^prog^ fates. The deletion of HDAC1 increased the accessibility of certain gene loci in early Tex^prog^ cells, as one might have expected from the well-known function of HDACs to promote a closed chromatin state (Seto and Yoshida, 2014; Yang and Seto, 2007). However, the integration of our ATAC-seq and scRNA-seq data did not show an enrichment in the expression of genes associated with these open loci in a particular subset, suggesting that HDAC1 does not restrain alternative fates of early Tex^prog^ cells. Unexpectedly, our analysis revealed that the deletion of HDAC1 resulted in a more closed chromatin state at gene loci in early Tex^prog^ cells that are later expressed in early Tex^eff-like^ cells. Since HDAC1 is recruited to many of these effector gene loci in WT early Tex^prog^ cells, this suggests a novel mode of action for HDAC1 in establishing an open chromatin state in early Tex^prog^ cells that facilitates differentiation toward early Tex^eff-like^ cells. Our study is in line with a recently published study (Hu et al., 2025) in which, by analyzing HDAC1-deficient (*Gzmb*-Cre) mice combined with scRNA-seq and ATAC-seq approaches, they showed that HDAC1 is an early determinant of intermediate-exhausted CD8^+^ T cell fate in chronic viral infection. Our observation that Runx3 is bound to many of the DARs that are open in WT early Tex^prog^ cells indicates that some of these gene loci might be co-regulated by HDAC1 and Runx3. Since Runx motifs were enriched in open DARs in both genotypes, this might also imply that Runx proteins might have different interaction partners and/or are recruited to different genomic loci in early Tex^prog^ cells dependent on the presence of HDAC1. Since members of the HDAC family target many nonhistone proteins (Choudhary et al., 2009; Ellmeier and Seiser, 2018), HDAC1 might also control the acetylation status and thus the functional properties of TFs key for the differentiation of Tex^eff-like^ cells. Our SCENIC analysis identified several regulons whose activities are increased in either WT or HDAC1-cKO Tex subsets. These regulons might represent interesting TFs to study whether their functions are regulated by HDAC1.

Seminal studies supported a linear model of Tex subset generation in which Tex^prog^, Tex^eff-like^, and Tex^term^ cell differentiation occurs sequentially (Beltra et al., 2020; Hudson et al., 2019). Subsequent studies using scRNA-seq combined with TCR-seq and trajectory analysis suggested a bifurcation model, where Tex^prog^ cells first differentiate into CX3CR1^+^ Tex^int^ cells, which then bifurcate into either Tex^eff-like^ (also known as Tex^KLR^) or Tex^term^ cells (Daniel et al., 2022; Fagerberg et al., 2025; Kasmani et al., 2023). We identified a CX3CR1-expressing cluster enriched with a Tex^int^ signature, and our trajectory analysis indicated that this Tex^int^ cluster differentiates into the Tex^eff-like^ cluster. Since the generation of the Tex^int^ cluster was severely impaired in the absence of HDAC1, this might lead to a differentiation defect of the Tex^eff-like^ cluster. Moreover, since HDAC1-cKO Tex^term^ cells (i.e., Tim3^hi^CD101^+^ cells) were reduced, it is conceivable that this alteration is also a consequence of impaired Tex^int^ cell subset differentiation. Therefore, we propose a model for HDAC1 function in which HDAC1 plays a key role for the transition of (early) Tex^prog^ to Tex^int^ cell subsets. HDAC1 deletion leads to a block in this transition, resulting in the expansion of the Tex^early^ cluster and their skewed differentiation into the Tex^cyt^ cluster. We also noticed that the Tex^prog^ cluster is separated into WT- and HDAC1-cKO–dominant subregions, suggesting the emergence of an early Tex^prog^ cell subset in the absence of HDAC1. It is therefore possible that these altered characteristics of early Tex^prog^ cells impact their later differentiation.

Our study has some limitations. Our data indicate an unexpected and novel role of HDAC1 in facilitating open chromatin at effector-like gene loci in early Tex^prog^ cells. However, HDACs in general not only function as epigenetic regulators, but reversible lysine acetylation also targets many nonhistone proteins. Thus, it will be important to address whether the effect observed on Tex^eff-like^ cells upon deletion of HDAC1 is (in part) due to changes in posttranslational modifications of factors driving Tex^eff-like^ differentiation. Moreover, while our data indicate a selective requirement of HDAC1 for Tex^eff-like^ cell subset differentiation, further studies are needed to elucidate how HDAC1 activity is modulated by multiple environmental cues to promote the formation of Tex^eff-like^ cell subset (Kanev and Zehn, 2021; Lan et al., 2023; Zander and Cui, 2023).

Pan-HDAC inhibitors are used for the treatment of certain types of cancers, and many show promising results in preclinical models for T cell–mediated and neurological diseases (Falkenberg and Johnstone, 2014). However, based on our findings, a careful assessment is required to evaluate the impact of such inhibitors, as they might impair the generation of Tex^eff-like^ subset. Conversely, our study suggests that the modulation of HDAC1 activity and the activation of HDAC1-controlled pathways might serve as an attractive strategy to improve the outcome of T cell–based immunotherapies by promoting the generation of Tex^eff-like^ cells.

## Materials and methods

### Mice

Floxed *Hdac1* (MGI:4440556) mice were kindly provided by Patrick Matthias (Friedrich Miescher Institute for Biomedical Research, Basel, Switzerland) and Christian Seiser (Medical University of Vienna, Vienna, Austria). *Cd4*-Cre mice (MGI:2386448) were kindly provided by Chris Wilson (University of Washington, Seattle, WA, USA). *Hdac1*^fl/fl^ and *Cd4*-Cre (HDAC1-cKO) mice were previously described (Grausenburger et al., 2010). *Rosa26*-STOP-YFP (MGI:2449038) and P14 T cell receptor transgenic (MGI:2665105) mice were kindly provided by Meinrad Busslinger (Research Institute of Molecular Pathology, Vienna, Austria) and Annette Oxenius (ETH Zurich, Zurich, Switzerland), respectively, and were crossed to HDAC1-cKO mice to generate either P14, *Hdac1*^fl/fl^, *Cd4*-Cre or *Rosa26*-STOP-YFP, P14, *Hdac1*^fl/fl^, and *Cd4*-Cre (P14-HDAC1-cKO or P14-HDAC1-cKO^YFP^, respectively) mice. *Rosa26*-CreERT2 mice (MGI:3699244) were kindly provided by Emilio Casanova (Medical University of Vienna, Vienna, Austria) and were crossed to *Hdac1*^fl/fl^ mice on a *Rosa26*-STOP-YFP background to generate *Rosa26*-STOP-YFP, *Hdac1*^fl/fl^, and *Rosa26*-CreERT2 (HDAC1-cKO^CreERT-YFP^) mice. CD45.1 (MGI:4819849) and CD90.1 (MGI:3579311) congenic mice were kindly provided by Jochen Hühn (Helmholtz Center, Braunschweig, Germany) and Annette Oxenius (ETH Zurich, Zurich, Switzerland), respectively. *GzmB*-Cre (MGI:2176191) mice were kindly provided by Susan Kaech (NOMIS Center for Immunobiology and Microbial Pathogenesis, Salk Institute, La Jolla, CA, USA) and were crossed to *Hdac1*^fl/fl^ mice on a *Rosa26*-STOP-YFP background to generate *Rosa26*-STOP-YFP, *Hdac1*^fl/fl^, and *GzmB*-Cre (HDAC1-cKO^Gzmb-YFP^). Mice were analyzed at 8–12 wk of age and were maintained in the Core Facility Laboratory Animal Breeding and Husbandry of the Medical University of Vienna. Animal experiments were assessed by the Ethics Committee of the Medical University of Vienna and approved by the Austrian Federal Ministry for Education, Science and Research (animal protocol number: BMBWF-66.009/0040-V/3b/2019, BMBWF-66.009/0343-V/3b/2019). Animal husbandry and experiments were performed in compliance with national laws and according to the Federation of European Laboratory Animal Science Association and the Animal Research: Reporting of In Vivo Experiments guidelines. Genotyping PCR primers are listed in Table S3.

### LCMV Cl13 infection and measurement of viral titer

LCMV Cl13 was propagated in BHK-21 cells (ATCC No. CCL-10) as described (Bergthaler et al., 2010; Welsh and Seedhom, 2008). Mice were infected i.v. through the tail vein with 2 × 10^6^ focus-forming units (FFUs) of LCMV Cl13. Mice were euthanized 8, 15, 22, 29, or 30 days after infection for further analysis. Viral titers were measured using standard immunofocus assays (Battegay et al., 1991) with minor modifications. Briefly, Vero cells (ATCC No. CCL-81) were incubated for 1 h with blood serum collected from infected mice. Afterward, the cells were covered for 48 h with 1x 199 media (Gibco) containing 0.5% medium electroendosmosis agarose (Biozym), 10% FBS (Biowest), 2 mM glutamine (Sigma-Aldrich), and 100 U/ml penicillin and streptomycin (Sigma-Aldrich). Cells were fixed with 25% formaldehyde (Sigma-Aldrich) in PBS (Sigma-Aldrich), permeabilized with 1% Triton X-100/PBS (Promega), and blocked with 5% FBS/PBS. Subsequently, cells were stained with VL-4 rat anti-LCMV nucleoprotein (BioXCell), followed by staining with anti-rat horseradish peroxidase (Jackson Immunoresearch) in 2% FBS/PBS. Infected cells were visualized using 3-amino-9-ethylcarbazole chromogen and substrate (Biolegend). Viral titers were calculated based on the number of colored “spots” on the plate and the dilution factor of the serum samples. Levels of serum alanine aminotransferase and aspartate aminotransferase were measured enzymatically (Roche Diagnostics).

### Generation of mixed BM chimeric mice

BM cells isolated from either WT or HDAC1-cKO mice (both expressing the congenic marker CD45.2) were mixed at a 1:1 ratio with BM cells from WT CD45.1 mice. Mixed BM cells (2 × 10^6^) were transferred i.v. into lethally irradiated CD45.1^+^ mice. Reconstituted mice were i.v. infected with 2 × 10^6^ FFUs of LCMV Cl13 7–8 wk after transplantation. Eight days later, mice were euthanized, and splenocytes were analyzed by flow cytometry.

### CD8^+^ T cell transfer followed by LCMV Cl13 infection

Naïve P14-WT and P14-HDAC1-cKO^YFP^ cells (CD90.2^+^) were isolated as described previously (Gülich et al., 2021) and mixed at a 1:1 ratio. Either 1 × 10^6^ (analyzed at 48 and 67 h p.i.) or 2 × 10^4^ (analyzed on day 5 and day 8 p.i.) of the mixed P14 cells were transferred i.v. into CD90.1^+^ congenic mice. One day after the transfer, recipient mice were infected i.v. with 2 × 10^6^ FFUs of LCMV Cl13. Mice were euthanized at various time points after infection as indicated, and splenocytes were analyzed using flow cytometry. For some co-transfer experiment, naïve P14-WT or P14-HDAC1-cKO cells (CD45.2^+^) were mixed at a 1:1 ratio with P14-WT-CD45.1 cells (CD45.1^+^CD45.2^+^) and adoptively transferred i.v. into CD90.1^+^ congenic mice. One day after the transfer, recipient mice were infected, subsequently analyzing splenocytes using flow cytometry at 8 days p.i.

### Tamoxifen treatment

For timed induction of *Hdac1* deletion, HDAC1-cKO^CreERT-YFP^ mice were treated with 2 mg of tamoxifen (Sigma-Aldrich) intraperitoneally after chronic disease has been established (i.e., 9 days p.i.). 6 days later (i.e., 15 days p.i.), mice were euthanized, and splenocytes were analyzed by flow cytometry.

### Flow cytometry analysis

Single-cell suspensions were prepared by mashing organs through a 70-µm cell strainer (Corning or pluriSelect). Hepatic lymphocytes were enriched via 35% Percoll (Cytiva)/RPMI 1640 (Sigma-Aldrich) gradient centrifugation (700 × *g* for 20 min at room temperature [RT], no breaks). Erythrocytes were lysed using 1× BD Pharm Lyse Buffer according to the manufacturer’s instruction (BD Biosciences). Cells were incubated with Fc block (1:250; BD Biosciences) and Fixable Viability Dye eFluor 506 (1:1,000; Thermo Fisher Scientific). For the detection of viral-specific CD8^+^ T cells, cells were subsequently incubated with fluorescently labeled H2-D^b^/GP33–41 tetramer (1:1,000; kindly provided by the NIH Tetramer Core Facility), followed by staining with antibodies for surface markers. Intracellular stainings were performed using the Foxp3/TF.

Staining Buffer Set (Thermo Fischer Scientific) according to the manufacturer’s instruction. For HDAC1 detection, cells were stained with primary rabbit anti-mouse HDAC1 antibody, followed by incubation with fluorescently labeled secondary goat anti-rabbit antibody. For intracytoplasmic detection of active caspase 3, splenocytes were incubated at 37°C for 5 h in complete RPMI1640 medium, supplemented with 10% FBS, 100 U/ml penicillin-streptomycin (Thermo Fisher Scientific), 2 mM L-glutamin (Thermo Fisher Scientific), 0.1 mM nonessential amino acid (Thermo Fisher Scientific), 1 mM sodium pyruvate (Sigma-Aldrich), and 55 μM of β-mercaptoethanol (Sigma-Aldrich). Subsequently, cells were fixed with BD Cytofix Fixation Buffer and were stained with Anti-Active Caspase-3 (BD Biosciences) in BD Perm/Wash Buffer (BD Biosciences) according to the manufacturer’s instructions (Beltra et al., 2020). For cytokine analysis, cells were stimulated with 1 µg/ml GP33–41 peptide, 0.7 μl/ml GolgiStop (BD Biosciences), and 1 μl/ml GolgiPlug (BD Biosciences) for 4.5 h. Cells were acquired with a BD LSRFortessa (BD Biosciences) or CytoFLEX (Beckman Coulter) and analyzed using FlowJo v10.8.1 software (BD Biosciences). Antibodies used in this study are listed in Table S4.

### Dimensionality reduction of flow cytometry data

Dimensionality reduction was performed using the DownSample and t-SNE Plugins in FlowJo v10.8.1 (BD Biosciences). For the generation of t-SNE plots, WT and HDAC1-cKO biological replicates were downsampled to the same number of cells/sample and concatenated for further analysis. t-SNE was run on the concatenated file using the default parameters provided by the software.

### scRNA-seq of naïve and virus-specific CD8^+^ T cells

Single-cell suspensions from the spleens of either LCMV-infected (infected as described above) or uninfected WT and HDAC1-cKO mice were prepared. Equal numbers of splenocytes from one female and one male mouse were combined for each scRNA-seq sample per condition. Samples were incubated with fluorescently labeled H2-D^b^/GP33–41 tetramer, appropriate antibodies against surface markers, oligonucleotide-conjugated antibodies (TotalSeq-A hashtags; Biolegend), and fixable viability dye (Zombie Green; Biolegend). Viable naïve (CD90.2^+^CD8α^+^CD44^−^) and LCMV-specific (CD90.2^+^CD8α^+^CD44^+^GP33-tet^+^) WT and HDAC1-cKO CD8^+^ T cells were sorted using an SH800S Cell Sorter (Sony Biotechnology). These four samples were pooled for multiplex sequencing (∼30,000 cells/run; a total of two runs was performed). Libraries were generated using the Chromium Controller and the Next GEM Single Cell 3′ Reagent Kit (v3.1, 10x Genomics) according to the manufacturer’s instructions. Libraries were sequenced by the Biomedical Sequencing Facility at CeMM using the Illumina NovaSeq 6000 platform. Raw sequencing results were converted to gene-barcode-count matrices using Cell Ranger (v6.1.2, 10x Genomics). The raw count matrices, which recovered 11,813 (first run) and 18,363 (second run) cells with 32,289 genes detected from both replicates, respectively, have been used for further computational analysis.

### Computational analysis of scRNA-seq data

Analysis was performed in R (version 4.1.2) using the Seurat package (version 4.2.0) as well as tidyverse (version 2.0.0), ggplot2 (version 3.4.2), and EnhancedVolcano (version 1.18.0). Replicates have been demultiplexed with a positive quantile threshold of 0.99 (HTOdemux() function) and resulted in a total of 7,108 negative, 2,398 doublet, and 20,670 singlet cells. Only singlets were used for cell type annotation with SingleR (version 1.8.1). All cells annotated as “T cells” or “NK cells” were kept (20,171 cells) for further analysis. The number of genes (nGene), number of unique molecular identifier (nUMI), percentage of mitochondrial genes (percent.mt), and the novelty score (number of genes detected per UMI [log_10_GenesPerUMI]) were used for quality control. Following filters were applied: 300 ≤ nGene ≤ 3,000, nUMI ≥ 500, percent.mt ≤ 5, and log_10_GenesPerUMI > 0.8. Before integrating the data from the two replicates, the 16,393 high-quality cells were used to regress out unwanted variations due to the expression of cell cycle genes (SCTransform() and CellCycleScoring()). Clustering was performed at different resolutions considering all principal components for which the change of variation to the subsequent principal component was larger than 0.1%. A clustering resolution of 0.6 was used for all following analyses and visualizations. Marker genes for all clusters were defined using FindAllMarkers() with the default settings. AddModuleScore() followed by FeaturePlot() was used to visualize the expression of previously published genes. Differentially expressed genes between groups were determined with FindMarkers(), where the minimum log_2_ fold change was set to 0 and the minimum fraction of cells in which a gene has to be detected in either of the two groups to be tested was set to 1%. GO term enrichment analyses were conducted with the packages clusterProfiler (version 4.8.1), org.Mm.e.g.,.db (version 3.17.0), and AnnotationHub (version 3.8.0). Top 200 marker genes of each cluster were used, and the resulting top five enriched pathways (in Biological Processes) from each cluster were collapsed to parent terms using Revigo (version 1.8.1) and then visualized with dotplot() from the enrichplot package (version 1.20.0). Trajectory inference was performed with Monocle3 (version 1.3.7), uniform manifold approximation and projection information was retrieved from clustering with Seurat, and cells were colored by pseudotime. Regulon activity analysis was conducted using SCENIC (version 1.3.1) in R using the final Seurat object as input. Regulon activity scores (area under the curve scores) were compared for each regulon between the groups using a Wilcoxon test followed by P value adjustment (false discovery rate [FDR]) using the stats package (version 4.5.0) and visualized as dotplots with the ggplot2 package.

### Sample preparation of virus-specific CD8^+^ T cells for ATAC-seq analysis

Naïve WT and HDAC1-cKO P14 T cells (CD90.2^+^) were isolated as described previously (Gülich et al., 2021), and 1 × 10^5^ cells were transferred i.v. into CD90.1^+^ congenic mice. One day after the transfer, recipient mice were infected i.v. with 2 × 10^6^ FFUs of LCMV Cl13. Eight days later, mice were euthanized, and splenocytes from 4 to 5 female mice were pooled for staining and subsequent cell sorting. 5 × 10^4^ early Tex^prog^ (CD90.2^+^CD8α^+^Tim3^−^Ly108^+^) and 5 × 10^4^ early non-Tex^prog^ (CD90.2^+^CD8α^+^Tim3^+^Ly108^−^) cells were sorted into PBS. High-throughput chromatin accessibility mapping (ATAC-seq) was performed as previously described (Buenrostro et al., 2013; Corces et al., 2016), with minor modifications. After centrifugation at 500 *g* for 5 min at 4°C, the cell pellets were resuspended in tagmentation-mix (containing Tagment DNA buffer, Tagment DNA Enzyme [llumina], Digitonin [Promega], and Proteinase K [Roche]) and incubated for 30 min at 37°C with shaking (300 rpm). The reaction was stopped by incubating the samples on ice. For DNA isolation, the MinElute PCR Purification Kit (Qiagen) was used according to the manufacturer’s instructions and eluted into 12 μl of elution buffer. 1 μl eluate was used in a quantitative PCR reaction to estimate the optimal number of cycles for library amplification. The remaining sample of each tagmented sample was then amplified corresponding to the C_q_ value (i.e., the cycle number at which fluorescence has increased above background levels) in the presence of custom Nextera primers. PCR amplification was followed by solid-phase reversible immobilization (SPRI) (Beckman Coulter) size selection to exclude fragments larger than 1,200 bp. The DNA concentration of each library was assessed with a Qubit 2.0 Fluorometric Quantitation system (Life Technologies) before pooling in equimolar amounts. The resulting pools were sequenced on a NovaSeq 6000 instrument (Illumina) in a 50-bp paired-end configuration. Chromatin accessibility mapping by ATAC-seq was done in three biological replicates. next generation sequencing (NGS) reads in unaligned BAM files were converted into FASTQ format with samtools, NGS adapter sequences were removed via fastp (0.23.2, 5′-GTC​TCG​TGG​GCT​CGG-3′), and the reads were aligned to the GRCm38 (UCSC Genome Browser mm10) assembly with Bowtie2 (2.4.5, --very-sensitive, --no-discordant, --maxins 2000) before deduplicating with samblaster (0.1.24). Alignment statistics and mitochondrial read fractions were collected with samtools. Peaks were called with MACS2 (2.2.7.1, --no-model, --keep-dup auto, --extsize 147), and peak annotation was performed with HOMER (4.11). UCSC Track hubs allowed for visual inspection of results. A consensus peak set was compiled, and all peaks in mm10 assembly regions blacklisted by the ENCODE project (Consortium, 2012) were removed. Sequencing and mapping analysis were performed by the Biomedical Sequencing Facility at CeMM. Resulting count matrices and peak files were used for further computational analysis.

### Computational analysis of ATAC-seq data

DARs in early Tex^prog^ and early non-Tex^prog^ were identified with the R (version 4.1.2) package DESeq2 (version 1.40.2) using the default log_2_ fold change threshold and an alpha of 0.05. Genes associated with DARs were used to calculate module scores (AddModuleScore() from the Seurat package [version 4.2.0]) to visualize the respective gene expression in the scRNA-seq clusters. For statistical testing, an ANOVA followed by Tukey’s test was performed. The same set of genes was also used to conduct the GO term enrichment analyses for biological processes with the same R packages as described above. The top 10 enriched pathways from each set were collapsed to parent terms using Revigo (version 1.8.1) and then visualized with dotplot() from the enrichplot package (version 1.20.0). Gene set enrichment analysis against a known effector signature (GSE9650_EFFECTOR_VS_EXHAUSTED_CD8_TCELL_UP) from the ImmuneSigDB was carried out using the packages msigdbr (version 7.5.1) and fgsea (version 1.26.0). TF motifs in DARs were determined by de novo motif analysis using findMotifsGenome.pl from HOMER (v4.10.3). Parameters were set to mask repeat regions (-mask), use the exact input region sizes (-size given), and find a maximum of 10 motifs per length (-S 10). P values <10^−11^ were considered true positive.

### Sample preparation of T cells for CUT&RUN analysis

WT and HDAC1-cKO mice infected with LCMV Cl13 were euthanized 8 days p.i,. and splenocytes from 10 mice per genotype were pooled. CD8^+^ T cells were enriched via REAlease CD8a MicroBead Kit mouse (Miltenyi Biotec) prior to staining and cell sorting. 1.5 × 10^6^ early Tex^prog^ (CD90.2^+^CD8α^+^Tim3^−^Ly108^+^) and 1.5 × 10^6^ early non-Tex^prog^ (CD90.2^+^CD8α^+^Tim3^+^Ly108^−^) cells were sorted into FCS. The sorted cells were pelleted at 400 × *g* for 5 min at 4°C, washed with PBS once, then resuspended in cold nuclear extraction buffer (20 mM HEPES [pH 7.9], 10 mM KCl, 0.1% Triton X-100, 20% glycerol, 1x cOmplete Mini-Tablet [Roche], 1 mM MnCl_2_, and 0.5 mM spermidine) and incubated for 10 min on ice. After centrifugation (400 × *g*, 5 min, 4°C), the nuclear pellet was resuspended in cold nuclear extraction buffer and kept on ice until ConA bead conjugation. ConA beads were prepared by washing twice with bead activation buffer (20 mM HEPES [pH 7.9], 10 mM KCl, 1 mM CaCl_2_, and 1 mM MnCl_2_) in a volume corresponding to 10x the original ConA bead volume and resuspended with bead activation buffer in the original volume. For CUT&RUN, 250,000 cells in 100 μl nuclear extraction buffer were incubated with 10-μl ConA beads for 10 min at RT. Nuclei-bound beads were then resuspended in 50 μl antibody buffer (20 mM HEPES [pH 7.5], 150 mM NaCl, 0.5 mM spermidine, 0.02% digitonin, 2 mM EDTA, and 1x cOmplete EDTA-free protease inhibitor cocktail [Roche]) containing 1 μl of H3K27ac antibody (Active Motif), rabbit IgG (Cell Signaling), H3K27me3 (Cell Signaling), HDAC1 (Cell Signaling), or Runx3 (Gift from Dr. Yoram Groner). Following overnight incubation at 4°C, beads were washed twice with 200 μl digitonin buffer (20 mM HEPES [pH 7.5], 150 mM NaCl, 0.5 mM spermidine, 0.02% digitonin, and 1x cOmplete EDTA-free protease inhibitor cocktail) and resuspended in 50 μl of digitonin buffer with pAG-MNase (IMP - Research Institute of Molecular Pathology). Beads were incubated for 30 min at RT, washed twice with 200 μl digitonin buffer, and MNase was activated as beads were resuspended in 50 μl digitonin buffer supplemented with 2 mM CaCl_2_ and incubated for 2 h at 4°C. The reaction was quenched upon addition of stop buffer (340 mM NaCl, 20 mM EDTA, 4 mM EGTA, 50 µg/ml RNaseA, and 50 mg/ml glycogen). Following incubation at 37°C for 10 min, the supernatant was transferred to a fresh tube. DNA was isolated with NEB Monarch PCR and DNA clean up. Libraries were prepared from 2 ng using NEBNext Ultra II DNA Library Prep Kit, cleaned up using 0.9x volume of SPRI beads, and subjected to PE150 sequencing on the Illumina NovaSeq 6000 platform.

### CTL assay

EL4 target cells were pulsed with 1 µg/ml of GP33–41 or OVA257–264 control peptides and labeled with CellTrace Violet to a final concentration of 5 µM or 0.5 µM, respectively. 1 × 10^4^ EL4 target cells were incubated for 4 h. with 0, 1 × 10^4^, 2 × 10^4^, or 4 × 10^4^ early non-Tex^prog^ (CD90.2^+^CD8α^+^Tim3^+^Ly108^−^) P14-WT or P14-HDAC1-cKO cells and afterward analyzed by flow cytometry.

### CUT&RUN bioinformatic analysis

Raw reads were aligned to the mm10 genome with bowtie2 (version 2.5.0). Blacklisted regions were removed via bedtools intersect (version 2.29.1). Duplicates were marked and, for control samples, removed with Picard tools MarkDuplicates (version 3.3.0-5-g46d23b4e4-SNAPSHOT). For quality control duplication rates, fragment lengths and replicate reproducibility were examined and plotted in R using the packages tidyverse (version 2.0.0) and ggplot2 (version 3.4.2). Reads, which mapped to mm10 as proper pairs, were merged to one BAM file per sample using samtools merge (version 1.21). RPKM normalized coverage files in BigWig format were generated with deeptools bamCoverage (version 3.5.6). Peaks were called with SEACR (version 1.3) for H3K27ac and H3K27me3 samples against IgG background and with MACS3 (version 3.0.3) for HDAC1 against KO background and Runx3 against IgG background samples. Peak calling resulted in BED or narrowPeak file output, respectively. These files together with the BigWig files were used for visualization of regions of interest in the IGV-Web app (version 2.2.7). Heatmaps and profiles were plotted using deeptools ComputeMatrix and plotHeatmap (version 3.5.6). Differential binding analysis was conducted with the R package Diffbind (version 3.18.0), setting summits = T. Numbers of open DARs from ATAC-seq overlapping with HDAC1 and Runx3-binding sites were determined using bedtools intersect (version 2.29.1). Volcano plots were done with the R package EnhancedVolcano (version 1.18.0). HOMER (v4.10.3) was used to annotate identified regions and to determine the most enriched TF motif in Runx3 peaks as described in the ATAC-seq analysis section.

### Statistical analysis

Statistical analysis was performed using Prism 8 or 9 (GraphPad). The values of the geometric mean fluorescence intensity were normalized by dividing the value of each sample by the average of the corresponding WT samples. Unpaired or paired two-tailed Student’s *t* test was used for comparisons between two groups. One-way ANOVA followed by Tukey’s multiple comparison test was used for the comparison of >2 groups. The P values are defined as follows: *P < 0.05; **P < 0.01; ***P < 0.001; n.s., not significant.

### Online supplemental material

Fig. S1 shows the percentages of Tex cell subsets in WT and HDAC1-cKO mice during LCMV infection and additional characterizations of early Tex cells 8 days p.i., related to Figs. 1, 2, and 3. Fig. S2 shows a series of experiments, related to Fig. 4, revealing a CD8^+^ T cell–intrinsic role for HDAC1 in Tex subset differentiation. Fig. S3 shows a further characterization of P14-WT and P14-HDAC1-cKO^YFP^ early Tex cells and early Tex subset distribution in WT^CreERT2^ and HDAC1-cKO ^CreERT2^ mice before tamoxifen administration, related to Figs. 5 and 6. Fig. S4 shows the supplemental data from the scRNA-seq analysis, related to Fig. 7. Fig. S5 shows the supplemental data from ATAC-seq and CUT&RUN analyses, related to Figs. 8 and 9. Table S1 shows the cell clusters and frequencies of early Tex cells as defined by scRNA-seq, related to Fig. 7. Table S2 shows the number of DARs in early Tex^prog^ and early non-Tex^prog^ cells as defined by ATAC-seq, related to Fig. 8. Table S3 shows the primer sequences used for genotyping experimental animals. Table S4 shows the list of antibodies used in this study.

## Supplementary Material

Table S1 shows the cell clusters and frequencies of LCMV-specific WT and HDAC1-cKO CD8^+^ T cells as defined by scRNA-seq.

Table S2 shows the number of DARs in WT and HDAC1-deficient early Tex^prog^ and early non-Tex^prog^ as defined by ATAC-seq.

Table S3 shows the primer sequences used for genotyping experimental animals.

Table S4 shows the antibody list.

## Data Availability

Raw and processed scRNA-seq, ATAC-seq, and CUT&RUN (sequence, count, and/or peak) data underlying this study are openly available in the NCBI GEO database with the following accession number: GSE255887. The subseries numbers are as follows: scRNA-seq: GSE255886, ATAC-seq: GSE255884, and CUT&RUN: GSE297018. Used code in form of scripts and relevant metadata underlying the analysis and visualization of the scRNA-seq, ATAC-seq, and CUT&RUN data are available in the author’s GitHub repository (https://github.com/medunivienna-IFI-immunobiology/2025_Rica-Waldherr-Miyakoda_HDAC1-CD8Tcells-chronicLCMV).
