# Supplementary material for: HDAC1 controls the generation and maintenance of effector-like CD8+ T cells during chronic viral infection
Source: J Exp Med. 2025 Jun 4;222(8):e20240829. doi: 10.1084/jem.20240829 (PMC12135962; doi:10.1084/jem.20240829)
Supplement: Table S1 — shows the cell clusters and frequencies of LCMV-specific WT and HDAC1-cKO CD8+ T cells as defined by scRNA-seq. [file jem_20240829_tables1.docx]

**Table S1: Cell clusters and frequencies of LCMV-specific WT and HDAC1-cKO CD8^+^ T cells as defined by scRNA-seq**

| **Cluster Number** | **Cluster Name** | **Genotype** | **Cell Number** | **Frequency of Genotype within Cluster** | **Frequency of Cluster within Genotype** |
| --- | --- | --- | --- | --- | --- |
| 1 | T^Naive^ | WT | 855 | 56% | 8.8% |
| 2 | Tex^prog^ | WT | 688 | 40% | 7.1% |
| 3 | Tex^prol^ | WT | 267 | 60% | 2.7% |
| 4 | Tex^exh^ | WT | 450 | 55% | 4.6% |
| 5 | Tex^early^ | WT | 404 | 13% | 4.1% |
| 6 | Tex^int^ | WT | 4,640 | 95% | 47.6% |
| 7 | Tex*^Cx3cr1^* | WT | 1,873 | 95% | 19.2% |
| 8 | Tex^cyt^ | WT | 572 | 28% | 5.9% |
| 1 | T^Naive^ | HDAC1-cKO | 664 | 44% | 10.0% |
| 2 | Tex^prog^ | HDAC1-cKO | 1,015 | 60% | 15.3% |
| 3 | Tex^prol^ | HDAC1-cKO | 176 | 40% | 2.6% |
| 4 | Tex^exh^ | HDAC1-cKO | 367 | 45% | 5.5% |
| 5 | Tex^early^ | HDAC1-cKO | 2,624 | 87% | 39.5% |
| 6 | Tex^int^ | HDAC1-cKO | 233 | 5% | 3.5% |
| 7 | Tex*^Cx3cr1^* | HDAC1-cKO | 93 | 5% | 1.4% |
| 8 | Tex^cyt^ | HDAC1-cKO | 1,472 | 72% | 22.2% |
