# Supplementary material for: HDAC1 controls the generation and maintenance of effector-like CD8+ T cells during chronic viral infection
Source: J Exp Med. 2025 Jun 4;222(8):e20240829. doi: 10.1084/jem.20240829 (PMC12135962; doi:10.1084/jem.20240829)
Supplement: Table S2 — shows the number of DARs in WT and HDAC1-deficient early Texprog and early non-Texprog as defined by ATAC-seq. [file jem_20240829_tables2.docx]

**Table S2: Number of DARs in WT and HDAC1-deficient early-Tex^prog^ and early-non-Tex^prog^ as defined by ATAC-seq**

| **Tex cell subset** | **Genotype** | **Annotation** | **Number of DARs** |
| --- | --- | --- | --- |
| Tex^prog^ | WT | intron | 40 |
| Tex^prog^ | WT | intergenic | 49 |
| Tex^prog^ | WT | promoter-TSS | 8 |
| Tex^prog^ | WT | exon | 2 |
| Tex^prog^ | WT | 5’ UTR | 0 |
| Tex^prog^ | WT | TTS | 1 |
| Tex^prog^ | WT | 3’ UTR | 0 |
| Tex^prog^ | WT | non-coding | 2 |
| Tex^prog^ | WT | total | 102 |
| Tex^prog^ | HDAC1-cKO | intron | 116 |
| Tex^prog^ | HDAC1-cKO | intergenic | 71 |
| Tex^prog^ | HDAC1-cKO | promoter-TSS | 14 |
| Tex^prog^ | HDAC1-cKO | exon | 5 |
| Tex^prog^ | HDAC1-cKO | 5’ UTR | 0 |
| Tex^prog^ | HDAC1-cKO | TTS | 2 |
| Tex^prog^ | HDAC1-cKO | 3’ UTR | 9 |
| Tex^prog^ | HDAC1-cKO | non-coding | 0 |
| Tex^prog^ | HDAC1-cKO | total | 217 |
| non-Tex^prog^ | WT | intron | 358 |
| non-Tex^prog^ | WT | intergenic | 290 |
| non-Tex^prog^ | WT | promoter-TSS | 29 |
| non-Tex^prog^ | WT | exon | 29 |
| non-Tex^prog^ | WT | 5’ UTR | 7 |
| non-Tex^prog^ | WT | TTS | 18 |
| non-Tex^prog^ | WT | 3’ UTR | 15 |
| non-Tex^prog^ | WT | non-coding | 3 |
| non-Tex^prog^ | WT | total | 749 |
| non-Tex^prog^ | HDAC1-cKO | intron | 618 |
| non-Tex^prog^ | HDAC1-cKO | intergenic | 447 |
| non-Tex^prog^ | HDAC1-cKO | promoter-TSS | 255 |
| non-Tex^prog^ | HDAC1-cKO | exon | 41 |
| non-Tex^prog^ | HDAC1-cKO | 5’ UTR | 23 |
| non-Tex^prog^ | HDAC1-cKO | TTS | 21 |
| non-Tex^prog^ | HDAC1-cKO | 3’ UTR | 18 |
| non-Tex^prog^ | HDAC1-cKO | non-coding | 7 |
| non-Tex^prog^ | HDAC1-cKO | total | 1430 |
