# Supplementary material for: HDAC1 controls the generation and maintenance of effector-like CD8+ T cells during chronic viral infection
Source: J Exp Med. 2025 Jun 4;222(8):e20240829. doi: 10.1084/jem.20240829 (PMC12135962; doi:10.1084/jem.20240829)
Supplement: Table S3 — shows the primer sequences used for genotyping experimental animals. [file jem_20240829_tables3.docx]

**Table S3: Primer sequences used for genotyping experimental animals**

| **Name** | **Sequence 5’-3’** |
| --- | --- |
| *Cd4*-Cre forward | TCTCTGTGGCTGGCAGTTTCTCCA |
| *Cd4*-Cre reversed | TCAAGGCCAGACTAGGCTGCCTAT |
| *Hdac1* forward | GGTAGTTCACAGCATAGTACTT |
| *Hdac1* reversed | CCTGTGTCATTAGAATCTACTT |
| P14-tg #1 | CATGGAGGCTGCAGTCACCC |
| P14-tg #2 | GTTTGTTTGCGAGCTCTGTTTTGATGGCTC |
| *Rosa26*-CreERT2 #1 | GTAACTGTGGACAGAGGAGCCATAAC |
| *Rosa26*-CreERT2 #2 | GGGAAACCATTTCCGGTTATTCAAC |
| *Rosa26*-STOP-YFP #1 | AAGACCGCGAAGAGTTTGTC |
| *Rosa26*-STOP-YFP #2 | AAAGTCGCTCTGAGTTGTTAT |
| *Rosa26*-STOP-YFP #3 | GGAGCGGGAGAAATGGATATG |
