# Supplementary material for: HDAC1 controls the generation and maintenance of effector-like CD8+ T cells during chronic viral infection
Source: J Exp Med. 2025 Jun 4;222(8):e20240829. doi: 10.1084/jem.20240829 (PMC12135962; doi:10.1084/jem.20240829)
Supplement: Table S4 — shows the antibody list. [file jem_20240829_tables4.docx]

**Table S4: Antibody list**

| **Epitope** | **Clone (or Cat. No.)** | **Company** |
| --- | --- | --- |
| anti-mouse 2B4 | m2B4 (B6)458.1 | Biolegend |
| anti-GFP (binding to YFP) | 1A12-6-18 | BD Biosciences |
| anti-mouse CD39 | Duha59 | Biolegend |
| anti-mouse CD45.1 | A20 | Biolegend |
| anti-mouse CD45.2 | 104 | Biolegend |
| anti-mouse CD69 | H1.2F3 | Thermo Fisher Scientific |
| anti-mouse CD8α | 53-6.7 | Biolegend |
| anti-mouse CD90.2 | 53-2.1 | Biolegend |
| anti-mouse CX3CR1 | SA011F11 | Biolegend |
| anti-mouse Eomes | Dan11mag | Thermo Fisher Scientific |
| anti-mouse Granzyme B | GB11 | Biolegend |
| rabbit anti-mouse HDAC1 | Sat13 | kindly provided by  Christian Seiser |
| anti-mouse Ki67 | 16A8 | Biolegend |
| anti-mouse Ly108 | 330-AJ | Biolegend |
| anti-mouse PD1 | 29F.1A12 | Biolegend |
| anti-mouse Perforin | s16009a | Biolegend |
| anti-mouse Tim3 | RMT3-23 | Biolegend |
| anti-mouse/human Active Caspase-3 | C92-605.rMAb | BD Biosciences |
| anti-mouse/human CD44 | IM7 | Biolegend |
| anti-mouse/human KLRG1 | 2F1/KLRG1 | Biolegend |
| anti-mouse/human T-bet | 4B10 | Biolegend |
| anti-mouse Tox | TXRX10 | Invitrogen |
| anti-mouse CD101 | Moushi101 | Thermo Fisher Scientific |
| anti-rat CD90/mouse CD90.1 | OX-7 | Biolegend |
| goat anti-rabbit IgG Alexa Fluor 647 | # ab150079 | Abcam |
| goat anti-rabbit IgG Alexa Fluor 488 | # A11008 | Thermo Fisher Scientific |
| H2-D^b^/GP33-41 tetramer | - | kindly provided by  NIH Tetramer Core Facility |
| TotalSeq™-A0301 anti-mouse Hashtag 1 | M1/42; 30-F11 | Biolegend |
| TotalSeq™-A0302 anti-mouse Hashtag 2 | M1/42; 30-F11 | Biolegend |
| TotalSeq™-A0303 anti-mouse Hashtag 3 | M1/42; 30-F11 | Biolegend |
| TotalSeq™-A0303 anti-mouse Hashtag 4 | M1/42; 30-F11 | Biolegend |
| H3K27ac | # 39133 | Active Motif |
| rabbit IgG | # 2729S | Cell Signaling Technology |
| H3K27me3 | C36B11 | Cell Signaling Technology |
| HDAC1 | 10E2 | Cell Signaling Technology |
| Runx3 | - | kindly provided by Yoram Groner |
